# Dissecting Herpes Simplex Virus 1-Induced Host Shutoff at the RNA Level

**DOI:** 10.1128/JVI.01399-20

**Published:** 2021-01-13

**Authors:** Caroline C. Friedel, Adam W. Whisnant, Lara Djakovic, Andrzej J. Rutkowski, Marie-Sophie Friedl, Michael Kluge, James C. Williamson, Somesh Sai, Ramon Oliveira Vidal, Sascha Sauer, Thomas Hennig, Arnhild Grothey, Andrea Milić, Bhupesh K. Prusty, Paul J. Lehner, Nicholas J. Matheson, Florian Erhard, Lars Dölken

**Affiliations:** a Institute of Informatics, Ludwig-Maximilians-Universität München, Munich, Germany; b Institute for Virology and Immunobiology, Julius-Maximilians-Universität Würzburg, Würzburg, Germany; c Department of Medicine, University of Cambridge, Cambridge, United Kingdom; d Cambridge Institute of Therapeutic Immunology and Infectious Disease, University of Cambridge, Cambridge, United Kingdom; e Max Delbrück Center for Molecular Medicine/Berlin Institute of Health, Berlin, Germany; f Helmholtz Institute for RNA-Based Infection Research (HIRI), Helmholtz Center for Infection Research (HZI), Würzburg, Germany; University of Arizona

**Keywords:** 4sU-seq, herpes simplex virus 1, RNA degradation, RNA-seq, chromatin-associated RNA, proteomics, transcriptional regulation, virion host shutoff protein

## Abstract

Herpes simplex virus 1 (HSV-1) induces a profound host shutoff during lytic infection. The virion host shutoff (*vhs*) protein plays a key role in this process by efficiently cleaving host and viral mRNAs. Furthermore, the onset of viral DNA replication is accompanied by a rapid decline in host transcriptional activity. To dissect relative contributions of both mechanisms and elucidate gene-specific host transcriptional responses throughout the first 8 h of lytic HSV-1 infection, we used transcriptome sequencing of total, newly transcribed (4sU-labeled) and chromatin-associated RNA in wild-type (WT) and Δ*vhs* mutant infection of primary human fibroblasts. Following virus entry, *vhs* activity rapidly plateaued at an elimination rate of around 30% of cellular mRNAs per hour until 8 h postinfection (p.i.). In parallel, host transcriptional activity dropped to 10 to 20%. While the combined effects of both phenomena dominated infection-induced changes in total RNA, extensive gene-specific transcriptional regulation was observable in chromatin-associated RNA and was surprisingly concordant between WT and Δ*vhs* infections. Both induced strong transcriptional upregulation of a small subset of genes that were poorly expressed prior to infection but already primed by H3K4me3 histone marks at their promoters. Most interestingly, analysis of chromatin-associated RNA revealed *vhs*-nuclease-activity-dependent transcriptional downregulation of at least 150 cellular genes, in particular of many integrin adhesome and extracellular matrix components. This was accompanied by a *vhs*-dependent reduction in protein levels by 8 h p.i. for many of these genes. In summary, our study provides a comprehensive picture of the molecular mechanisms that govern cellular RNA metabolism during the first 8 h of lytic HSV-1 infection.

**IMPORTANCE** The HSV-1 virion host shutoff (*vhs*) protein efficiently cleaves both host and viral mRNAs in a translation-dependent manner. In this study, we model and quantify changes in *vhs* activity, as well as virus-induced global loss of host transcriptional activity, during productive HSV-1 infection. In general, HSV-1-induced alterations in total RNA levels were dominated by these two global effects. In contrast, chromatin-associated RNA depicted gene-specific transcriptional changes. This revealed highly concordant transcriptional changes in WT and *Δvhs* infections, confirmed DUX4 as a key transcriptional regulator in HSV-1 infection, and identified *vhs*-dependent transcriptional downregulation of the integrin adhesome and extracellular matrix components. The latter explained seemingly gene-specific effects previously attributed to *vhs*-mediated mRNA degradation and resulted in a concordant loss in protein levels by 8 h p.i. for many of the respective genes.

## Abstract (precis)

The HSV-1 virion host shutoff (*vhs*) protein efficiently cleaves both host and viral mRNAs in a translation-dependent manner. In this study, we model and quantify changes in *vhs* activity, as well as virus-induced global loss of host transcriptional activity, during productive HSV-1 infection. In general, HSV-1-induced alterations in total RNA levels were dominated by these two global effects. In contrast, chromatin-associated RNA depicted gene-specific transcriptional changes. This revealed highly concordant transcriptional changes in WT and *Δvhs* infections, confirmed DUX4 as a key transcriptional regulator in HSV-1 infection, and identified *vhs*-dependent transcriptional downregulation of the integrin adhesome and extracellular matrix components. The latter explained seemingly gene-specific effects previously attributed to *vhs*-mediated mRNA degradation and resulted in a concordant loss in protein levels by 8 h p.i. for many of the respective genes.

## INTRODUCTION

Herpes simplex virus 1 (HSV-1), one of eight herpesviruses infecting humans, is widely known for causing cold sores but also associated with life-threatening diseases such as encephalitis (1, 2). A key characteristic of HSV-1 lytic infection is the induction of a profound host shutoff that is predominantly induced at the RNA level. The virion host shutoff (*vhs*) endonuclease plays a crucial role in this process. *vhs* is delivered by the tegument of the incoming virus particles and, together with *de novo*-expressed *vhs* protein, rapidly starts cleaving both cellular and viral mRNAs in a translation-initiation-dependent manner (3–8). Later on in infection, *vhs* nuclease activity is dampened by the concerted action of at least two viral proteins, i.e., UL48 (VP16) and UL49 (VP22) (9–11), with the viral UL47 protein (VP13/14) potentially also being involved (12). In addition to *vhs*-mediated mRNA degradation, HSV-1 shuts down host gene expression by efficiently recruiting RNA polymerase II (Pol II) and elongation factors from the host chromatin to the replicating viral genomes (13–15). This results in an extensive loss of Pol II occupancy from host chromatin starting with the advent of viral DNA replication by 2 to 3 h postinfection (p.i.) (16). Furthermore, HSV-1 induces proteasome-dependent degradation of Pol II later (>12 h p.i.) in infection (17). Finally, extensive RNA degradation upon cleavage by the *vhs* nuclease also appears to contribute to the transcriptional shutoff by 24 h p.i. (18).

**FIG 1:**
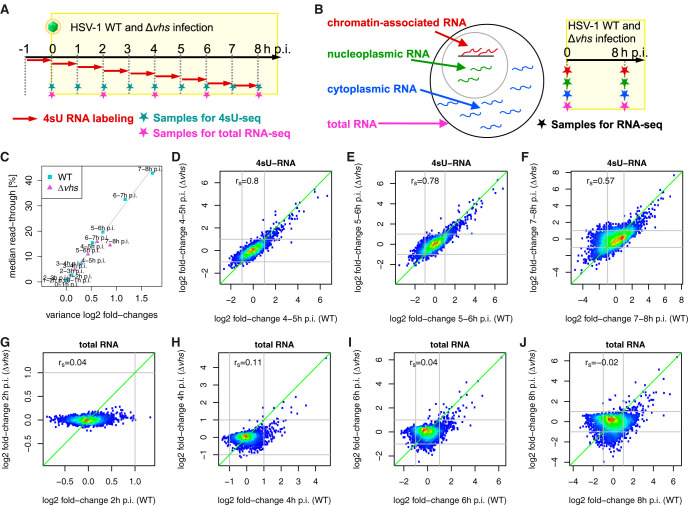
Experimental setup and correlation of gene expression changes. (A and B) Experimental setup of the 4sU-seq and total RNA time courses (A) and sequencing of subcellular RNA fractions (B) in HSV-1 WT and *Δvhs* infections. The time course experiments for the two viruses were performed as two independent experiments. Infections for the subcellular RNA fractions were performed within the same experiment. Data for WT infection for both experiments have already been published (20, 21). (C) Median read-through values (*y* axis) are linearly correlated to variance in log_2_-fold change (*x* axis) for the 4sU-seq time courses in WT (cyan) and Δ*vhs* (purple) infections. The gray line indicates a linear fit to WT samples. (D to J) Comparison of log_2_-fold change in gene expression (infected versus mock infected) between WT infection (*x* axis) and Δ*vhs* infection (*y* axis) for 4sU-seq RNA from 4 to 5 h (4–5 h) p.i. (D), 5–6 h p.i. (E), and 7–8 h p.i. (F), as well as for total RNA from 2 h (G), 4 h (H), 6 h (I), and 8 h (J) p.i. Points are color-coded according to density of points: from red = high density to blue = low density. The Spearman rank correlation *r_S_* is shown at the top left of each panel.

Both *vhs*-mediated mRNA degradation and global inhibition of transcription substantially alter the host transcriptome during productive infection. Virus-induced alterations in total RNA levels can be a consequence of either of these two global phenomena or due to gene-specific changes in RNA stability or transcription. Their relative contributions, however, could so far not be distinguished. Recently, Pheasant et al. presented a genome-scale transcriptome sequencing (RNA-seq) study analyzing nuclear-cytoplasmic compartmentalization of viral and cellular transcripts during lytic HSV-1 infection (19). These researchers proposed that the translational shutoff induced by HSV-1 is primarily a result of *vhs*-induced nuclear retention and not degradation of infected cell mRNA. Furthermore, they suggested differential susceptibility of transcripts to *vhs* RNA cleavage activity. We previously performed 4-thiouridine (4sU) labeling, followed by sequencing (4sU-seq), to characterize *de novo* transcription and RNA processing in hourly intervals during the first 8 h of lytic HSV-1 infection of primary human foreskin fibroblasts (HFF) (Fig. 1A) (20, 21). This revealed extensive poly(A) read-through transcription into downstream intergenic regions resulting from disruption of transcription termination (DoTT) for the majority of, but not all, cellular genes. Due to nuclear retention of the respective aberrant transcripts, DoTT also notably contributes to host shutoff (21). Furthermore, read-in transcription from upstream genes commonly results in the seeming induction of genes. DoTT and read-in transcription thus confound the analysis of changes in cellular RNA levels and host transcriptional activity during HSV-1 infection.

To dissect the effects of *vhs*-mediated RNA degradation and global loss in transcriptional activity during lytic HSV-1 infection on a genome-wide scale, we now performed total RNA-seq and 4sU-seq time course analysis on HFF infected with a *vhs*-null mutant virus in which *vhs* has been inactivated by replacement of amino acids 251 to 489 with LacZ (Δ*vhs*) (22). Here, we used the same experimental setting and standardized conditions as previously employed for wild-type (WT) HSV-1 infection (Fig. 1A) (20). Furthermore, we analyzed subcellular RNA fractions (cytoplasmic, nucleoplasmic, and chromatin-associated RNA) at 0 and 8 h p.i. of WT and Δ*vhs* infections (Fig. 1B). Mathematical modeling of RNA synthesis and *vhs*-mediated RNA decay revealed that *vhs* activity rapidly plateaued upon WT HSV-1 infection with *vhs* continuously degrading about 30% of cellular mRNAs per hour until at least 8 h p.i. In contrast, total RNA changes in Δ*vhs* infection were dominated by the global loss in Pol II activity. Changes in total mRNA levels upon HSV-1 infection are thus shaped by differences in basal transcription and RNA turnover rates between the individual genes. In contrast, chromatin-associated RNA provided an unbiased picture of gene-specific transcriptional changes. This revealed an extensive, previously unsuspected *vhs*-dependent transcriptional downregulation of the integrin adhesome and extracellular matrix (ECM). Notably, this included the key *vhs*-sensitive genes reported by Pheasant et al. Accordingly, increased reduction of total mRNA levels for these genes is not due to increased susceptibility to *vhs*-mediated RNA decay of the respective transcripts but instead due to additional, *vhs*-cleavage-activity-dependent effects on their transcription. *vhs*-dependent downregulation of transcriptional activity resulted in reduced protein levels of many of the respective genes already at 8 h p.i. in WT infection but not in *Δvhs* infection, as confirmed by quantitative whole-proteome mass spectrometry.

## RESULTS

### Genome-wide RNA-seq analysis in WT and Δ*vhs* infection.

To dissect the role of *vhs*, global inhibition of Pol II activity and host gene-specific regulation during productive HSV-1 infection, we employed the same experimental set-up for Δ*vhs* infection as for our previous transcriptome analyses on WT HSV-1 infection (20). We infected HFF with Δ*vhs* at a high multiplicity of infection (MOI) of 10 and performed 4sU-seq in hourly intervals and total RNA-seq every 2 h during the first 8 h of infection (two biological replicates; Fig. 1A). Consistent with our previous findings (20, 23) and with the modest attenuation of the Δ*vhs* mutant in HFF, HSV-1-induced DoTT affected the same genes in Δ*vhs* infection but was less prominent compared to WT infection (Fig. 1C; see also Data Set S1 in the supplemental material). Since read-in transcription into downstream genes due to HSV-1-induced DoTT from upstream genes can be mistaken for “induction” of these downstream genes (20), we excluded genes with read-in transcription from all following analyses (see Materials and Methods for details). This resulted in a set of 4,162 genes without read-in transcription, for which RNA fold changes comparing infection versus mock infection and their significances were determined using DESeq2 (24). Unless otherwise noted, all fold changes indicated below are always made in comparison to mock infection from the corresponding experiments.

DESeq2 normalization assumes that there are no global changes in RNA levels between conditions. This is not the case in HSV-1 infection due to *vhs*-mediated RNA degradation and the global loss of transcriptional activity. A possible approach to normalize to decreasing RNA levels during infection uses RNA spike-ins (19, 25, 26). However, such a normalization effectively only decreases the fold changes by a constant factor (or a constant additive term for log_2_-fold change) for all genes. It does not affect the correlation between fold changes (evaluated as the Spearman rank correlation *r_S_* below), which is scale invariant. Since the analyses here were all performed without such normalization, the fold changes discussed here always represent relative changes compared to other genes regarding their relative contribution to overall RNA levels. Accordingly, this means that genes identified here as nonregulated only exhibit the global reductions in RNA levels or transcription that equally affect all genes. This enables the identification of gene-specific changes in RNA abundance and transcription levels that differ from the majority of genes.

### Delineating *vhs*-mediated RNA degradation and loss of transcriptional activity.

Gene expression fold changes in 4sU-RNA were highly correlated between Δ*vhs* and WT infections when comparing the same time points, confirming the high degree of standardization between the two independent experiments (Fig. 1D to F). The only exceptions were the first two 4sU-seq time points (0–1 and 1–2 h p.i., Spearman rank correlation [*r_S_*] ≤ 0.3), when essentially no (*n* ≤ 2) cellular genes were differentially expressed in both WT and Δ*vhs* infections (multiple testing adjusted *P* ≤ 0.001, |log_2_-fold change| ≥ 1). This was expected as fold changes were only very small (median |log_2_-fold change| ≤ 0.1) and dominated by experimental noise. The highest correlations between 4sU-seq fold changes in WT and Δ*vhs* infections compared to mock infections were observed at 4–5 h and 5–6 h p.i. (*r_S_* ≈0.8, Fig. 1D and E). Correlations decreased toward the end of the time course in particular for genes downregulated in WT (Fig. 1F), consistent with the well described effects of *vhs* on cellular RNA levels late in infection (27). Notably, the later stages of Δ*vhs* infection (from 6–7 h p.i.) were better correlated with slightly earlier stages (4–5 h p.i. and 5–6 h p.i.) of WT infection, indicating a slightly slower progression of Δ*vhs* infection.

**FIG 2:**
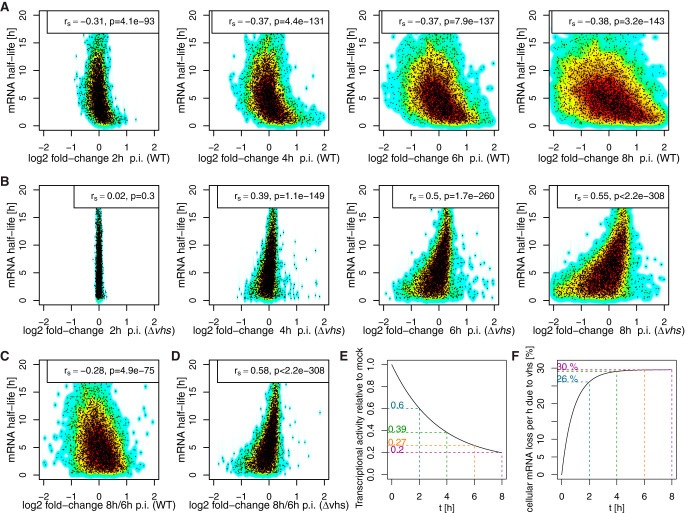
Effects of *vhs* activity and loss of transcriptional activity. (A and B) Comparison of log_2_-fold change in total RNA at 2, 4, 6, and 8 h p.i. versus mock infection (*x* axis), respectively, against RNA half-lives (*y* axis) for WT (A) and Δ*vhs* (B) infections. Background indicates the density of points: from dark red = high density to cyan = low density. The Spearman rank correlation *r_S_* and the *P* value for the significance of correlation are shown at the top of each panel. (C and D) Comparison of log_2_-fold change in total RNA at 8 h p.i. versus 6 h p.i. (*x* axis) against RNA half-lives (*y* axis) for WT (C) and Δ*vhs* (D) infections. Negative correlations for WT infection indicate ongoing *vhs* activity between 6 and 8 h p.i. Positive correlations for Δ*vhs* infection are indicative of increasing loss of transcription during this time. (E) Decrease in transcriptional activity relative to uninfected cells (*y* axis) during HSV-1 infection (*x* axis = h p.i.) estimated with our mathematical model from total RNA-seq data in Δ*vhs* infection (see Text S2 in the supplemental material). (F) Development of *vhs* activity over time as estimated with our mathematical model from total RNA-seq data in WT infection (assuming the same decrease in transcriptional activity as for Δ*vhs* infection, see Text S2). The *x* axis indicates h p.i., and the *y* axis shows the rate of cellular mRNA loss per hour (as a percentage) due to *vhs* activity.

In contrast to 4sU-RNA, fold changes in total RNA obtained from WT and Δ*vhs* infection were only poorly correlated (*r_S_* ≤ 0.11, Fig. 1G to J). Consistent with the cleavage activity of *vhs*, this was particularly prominent for genes downregulated in WT infection. Since 4sU-RNA was purified from total RNA, the poor correlation for total RNA fold changes cannot be explained by poor reproducibility between the two independent experiments. We conclude that this instead reflects the expected strong impact of *vhs* cleavage activity on the cellular mRNAs. In principle, *vhs* cleavage activity should more strongly affect total mRNA levels of long-lived mRNAs than of short-lived mRNAs, since the former have much weaker *de novo* transcription relative to total RNA levels and are thus much more slowly replaced. On the contrary, HSV-1-induced global loss in transcriptional activity should more strongly affect total RNA levels of unstable, short-lived mRNAs. To test this hypothesis, we correlated the observed changes in total RNA upon WT and Δ*vhs* infections with RNA half-lives of the respective transcripts in uninfected cells. RNA half-lives for all analyzed genes were measured in uninfected HFF based on newly transcribed to total RNA ratios as previously described (see also Materials and Methods) (28). Please note that in the following, we always refer to basal mRNA half-lives in uninfected cells, not during infection. This correlation analysis revealed the expected striking differences between WT and Δ*vhs* infections. In WT infection, total RNA fold changes and mRNA half-lives were negatively correlated (*r_S_* = –0.38 at 8 h p.i., Fig. 2A), i.e., total RNA levels of stable cellular mRNAs tended to decrease more strongly than of unstable mRNAs. This was already observable at 2 h p.i. (*r_S_* = –0.31), consistent with mRNA cleavage and degradation by tegument-delivered *vhs* protein. The negative correlation to RNA half-lives was also confirmed for total RNA fold changes from the study of Pheasant et al. at 4 h p.i. (*r_S_* = –0.36), while at 12 h p.i., a weaker, but still highly significant, negative correlation was observed (*r_S_* = –0.15).

In Δ*vhs* infection, however, total RNA fold changes and RNA half-lives were positively correlated from 4 h p.i. onward (*r_S_* = 0.55 at 8 h p.i., Fig. 2B). Thus, total RNA levels of short-lived cellular RNAs were more strongly reduced than of long-lived ones. This effect is consistent with the well-described gradual decline in global transcriptional activity starting around 3 to 4 h p.i. (15, 20). Accordingly, total RNA fold changes in Δ*vhs* infection largely reflect the global loss in transcriptional activity during lytic HSV-1 infection rather than gene-specific regulation. The presence of negative correlations in WT infection, however, suggests that *vhs*-mediated RNA decay, not the global reduction in transcriptional activity on cellular genes, dominates total RNA fold changes in lytic WT HSV-1 infection. Although a correlation of –0.38 may not appear high, it is surprisingly strong considering that the loss of transcriptional activity that is also present in WT infection would lead to a positive, i.e., opposite, correlation without *vhs* activity. Interestingly, negative correlations in WT infection and positive correlations in Δ*vhs* infection to RNA half-lives were also observed for total RNA fold changes between 2 and 4 h p.i., 4 and 6 h p.i. and 6 and 8 h p.i. (Fig. 2C and D). *vhs* cleavage activity thus continues to dominate changes in total RNA levels at least until 8 h p.i. Nevertheless, the much weaker negative correlation at 12 h p.i. observable in the data of Pheasant et al. are consistent with a nearly complete loss of *vhs*-mediated cleavage activity at later times of infection by the combined action of the viral VP16 and VP22 proteins (9–11).

To estimate the kinetics of *vhs* activity, we developed an ordinary differential equation (ODE) model of HSV-1 infection that models (i) global changes in host transcriptional activity, (ii) global changes in *vhs* endonuclease activity, (iii) subcellular compartmentalization and nuclear export of transcripts, (iv) differences in basal mRNA half-lives between genes, and (v) gene-specific transcriptional regulation (see Text S2 in the supplemental material). Using this model, we estimated the extent of loss in transcriptional activity from our total RNA-seq time course data in Δ*vhs* infection and the increase of *vhs* endonuclease activity during WT HSV-1 infection from our total RNA time course in WT infection (see Text S2). Our results indicate that by 8 h p.i. in Δ*vhs* infection, transcriptional activity dropped down to 10 to 20% of the level in uninfected cells (Fig. 2E). Assuming an at least similar decrease in transcriptional activity in WT infection, our model suggests that at the height of *vhs* activity, ∼30% of cellular RNA molecules are lost per hour due to *vhs*-mediated RNA degradation (Fig. 2F). This rate reached 26% as early as 2 h p.i. and remained fairly constant until 8 h p.i. It is important to note that our data exclude a significant drop in *vhs* activity until 8 h p.i., since the decrease in transcriptional activity would otherwise have resulted in positive correlations between total RNA fold changes and mRNA half-lives in WT infection (see Text S2). Furthermore, if the loss of transcriptional activity in WT infection were indeed dramatically higher than in Δ*vhs* infection, *vhs*-mediated degradation would have to increase even faster and to higher levels to achieve the observed negative correlations. In summary, our model explains the wide range of total RNA fold changes observed between genes in HSV-1 infection simply by differences in basal RNA half-lives between genes in uninfected cells and gene-specific transcriptional regulation. Although statistically significant correlations were also observed between 4sU-RNA fold changes and RNA half-lives, these were relatively small in both WT (*r_S_* ≥ –0.15) and Δ*vhs* (*r_S_* ≤ 0.25) infections. Thus, changes in newly transcribed RNA obtained during 60 min of 4sU labeling are also influenced by *vhs*-mediated decay and loss of transcriptional activity but substantially less strongly than for total RNA. We conclude that the poor correlation in total RNA fold changes between WT and Δ*vhs* infections is a direct consequence of global effects of *vhs* on RNA stability throughout the first 8 h of lytic infection.

### Chromatin-associated RNA allows unbiased quantification of transcriptional regulation during HSV-1 infection.

**FIG 3:**
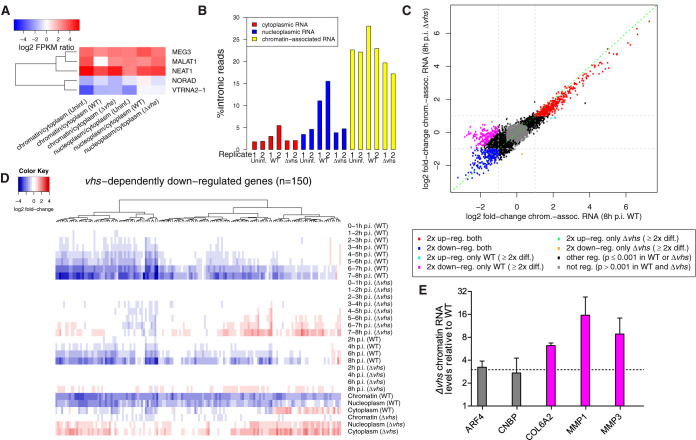
Transcriptional changes in WT and Δ*vhs* infections. (A) Log_2_ gene expression (FPKM) ratios for nucleoplasmic versus cytoplasmic RNA and chromatin-associated versus cytoplasmic RNA for three well-described nuclear lincRNAs (MEG3, MALAT1, and NEAT1) and two cytoplasmic lincRNAs (NORAD and VTRNA2-1). (B) Percentage of intronic reads [100 × no. of intronic reads/(no. of intronic reads + no. of exonic reads)] for cytoplasmic, nucleoplasmic, and chromatin-associated RNA in mock, WT, and Δ*vhs* infections shows an enrichment of intronic reads in chromatin-associated RNA. Parts of this figure for mock and WT infections were also shown in previous publications (21, 88). (C) Comparison of log_2_-fold change in chromatin-associated RNA at 8 h p.i. between WT (*x* axis) and Δ*vhs* (*y* axis) infections. Genes up- (log_2_-fold change ≥ 1, adj. *P* ≤ 0.001) or downregulated (log_2_-fold change ≤ –1, adj. *P* ≤ 0.001) in both WT and Δ*vhs* infections are indicated in red and blue, respectively. Genes transcriptionally downregulated in a *vhs*-dependent manner (log_2_-fold change ≤ −1, adj. *P* ≤ 0.001 in WT; log_2_-fold change > −1 in Δ*vhs* infection, as well as >2-fold difference in regulation) are marked in magenta. (D) Heatmap of log_2_-fold change in 4sU-RNA, total RNA, and subcellular RNA fractions in WT and Δ*vhs* infections for *vhs*-dependently downregulated genes (magenta in panel C). Genes were clustered according to Euclidean distances and Ward’s clustering criterion (see Materials and Methods). (E) qRT-PCR measurements for relative transcription of *vhs*-dependently downregulated genes COL6A2, MMP1, and MMP3 and control genes ARF4 and CNBP, which exhibit no gene-specific regulation, in Δ*vhs*-infected versus WT-infected cells at 8 h p.i. Means from *n* = 2 replicates are plotted with standard deviations as error bars.

Since our analysis revealed some effect of *vhs*-mediated decay and loss of transcriptional activity on 4sU-RNA, we analyzed subcellular RNA fractions (cytoplasmic, nucleoplasmic, and chromatin-associated RNA) from mock-, WT-, and Δ*vhs*-infected cells at 8 h p.i. (*n* = 2; Fig. 1B) to obtain an unbiased picture of transcriptional activity in WT and Δ*vhs* infection. Here, subcellular fractions for mock-, WT-, and Δ*vhs*-infected cells were obtained and sequenced in the same experiment. Only the data from mock- and WT-infected cells have previously been published (21). Known nuclear lincRNAs (MEG3, MALAT1, and NEAT1) were enriched in nucleoplasmic and chromatin-associated RNA, and cytoplasmic lincRNAs (NORAD and VTRNA2-1) were enriched in cytoplasmic RNA (Fig. 3A), confirming the efficient separation of the cytoplasmic and nuclear RNA fractions. Efficient separation of chromatin-associated RNA from nucleoplasmic RNA was confirmed by the strong overrepresentation of intronic reads in chromatin-associated RNA (Fig. 3B). Note that the increase in intronic reads in the nucleoplasmic RNA fraction in WT infection is due to extensive poly(A) read-through, which results in read-in transcription into downstream genes coupled with incomplete splicing and nuclear retention of read-through transcripts (20, 21). This was also observed in Δ*vhs* infection; however, it was less pronounced, consistent with the reduced levels of read-through transcription. Of note, the subcellular RNA fraction experiment also comprised total cellular RNA samples from WT and Δ*vhs* infections. The total RNA fold changes here nicely matched the 8-h time point in the respective WT (*r_S_* = 0.73) and Δ*vhs* (*r_S_* = 0.82) time courses. This also confirmed the poor correlation of total RNA fold changes between WT and Δ*vhs* infections (*r_S_* = 0.24). Thus, it does not result from experimental bias between two independently performed time course experiments. Furthermore, negative (*r_S_* = –0.36) and positive (*r_S_* = 0.34) correlations to RNA half-lives were again observed for WT and Δ*vhs* infections, respectively.

Since chromatin-associated RNA remains attached to the chromatin by the actively transcribing polymerases, it should not be accessible to *vhs*-mediated RNA cleavage and degradation. The absence of any significant correlation between fold changes in chromatin-associated RNA and RNA half-life for both WT and Δ*vhs* infections (*r_S_*=–0.08 for WT infection and 0.07 for Δ*vhs* infection) confirms this assumption and provides further evidence for the efficient separation of the chromatin-associated RNA fraction. We thus focused on changes in chromatin-associated RNA to assess the effects of HSV-1 infection and *vhs* on transcriptional regulation. Strikingly, comparison of chromatin-associated RNA fold changes revealed that changes in relative, gene-specific transcriptional activity at 8 h p.i. were extremely similar between WT and Δ*vhs* infections (*r_S_* = 0.89, Fig. 3C). Thus, although the global loss in transcriptional activity is higher in WT than Δ*vhs* infections due to a slower progression of Δ*vhs* infection, gene-specific regulation of transcriptional activity for individual genes remains mostly the same. The only exception was a set of 150 genes which were transcriptionally downregulated (beyond the general loss of transcriptional activity) only in WT infection and not in Δ*vhs* infection (magenta in Fig. 3C). These are further analyzed below.

Notably, 4sU-RNA fold changes were better correlated with fold changes in chromatin-associated RNA (*r_S_* ≈0.76) than to nucleoplasmic (*r_S_* ≈0.68) or cytoplasmic (*r_S_* ≈0.53) RNA, whereas the total RNA fold changes were best correlated with cytoplasmic RNA changes (*r_S_* ≈0.74). This indicates that even with a relatively long 4sU-labeling duration of 60 min, 4sU-RNA to a large degree represents ongoing nascent transcription on the chromatin level. We conclude that fold changes in chromatin-associated RNA provide an unbiased picture of transcriptional regulation in both WT and Δ*vhs* infections.

### *vhs*-dependent transcriptional downregulation of the extracellular matrix and integrin adhesome.

Differential gene expression analysis on chromatin-associated RNA identified 225 genes (5.4% of all genes) that were significantly downregulated at the transcriptional level (log_2_-fold change ≤ –1, adjusted [adj.] *P ≤* 0.001) in both WT and Δ*vhs* infections compared to mock infection (blue in Fig. 3C). This means that these downregulated genes—such as genes only downregulated in WT infection (magenta in Fig. 3C)—show even further reductions in transcription rates than nonregulated genes (gray and black in Fig. 3C). Notably, for the latter genes overall transcription rates also decrease but only due to the general loss of host transcriptional activity in infection, and they thus show no apparent gene-specific regulation. The concordantly downregulated genes (blue in Fig. 3C) were characterized by lower poly(A) read-through than non- or upregulated genes. Thus, their increased downregulation cannot be explained by negative effects of poly(A) read-through transcription on gene expression. Gene Ontology (GO) (29) enrichment analysis for these genes did not yield any statistically significant results. However, when comparing these genes to the INTERFEROME database (30), we observed significant enrichment (adj. *P* ≤ 0.001) for genes downregulated by type II interferon.

Interestingly, a set of 150 genes (3.6% of all genes) was significantly downregulated (log_2_-fold change ≤-1, adj. *P* ≤0.001) in WT infection but not in Δ*vhs* infection (marked magenta in Fig. 3C; see Data Set S3). *vhs*-dependent downregulation of these genes was confirmed in nucleoplasmic RNA, 4sU-RNA from 6–7 h p.i. onward, and in parts also in total RNA from 6 h p.i. onward (Fig. 3D). To validate *vhs*-dependent transcriptional downregulation by quantitative reverse transcription-PCR (qRT-PCR), we harvested chromatin-associated RNA at 8 h p.i. in WT and Δ*vhs* infections (*n* = 2 additional replicates, Fig. 3E). The relative RNA levels in Δ*vhs* infection versus WT infection were measured for two genes (control) that showed no gene-specific regulation (ARF4 and CNBP, from the gray genes in Fig. 3C) and two genes with *vhs*-dependent transcriptional downregulation (COL6A2 and MMP1, from the magenta genes in Fig. 3C). In addition, we included MMP3 since it was one of the genes whose strong reduction in total RNA levels in HSV-1 infection was shown to be *vhs* dependent by Pheasant et al. (19). Although MMP3 was not included in our primary analysis due to its proximity to nearby genes, differential gene expression analysis for all human genes on chromatin-associated RNA showed that MMP3 was also transcriptionally downregulated in a *vhs*-dependent manner (see Data Set S4 for the extended set of 578 *vhs*-dependent genes). Like Pheasant et al., we used 18S rRNA as an internal reference for the qRT-PCR since it is not translated and thus not targeted by *vhs*. As expected, ARF4 and CNBP both showed higher (∼3-fold) chromatin-associated RNA levels in Δ*vhs* infection than in WT infection. This is consistent with slower progression of Δ*vhs* infection compared to WT infection and thus reduced global reduction in host transcriptional activity. Nevertheless, the loss of COL6A2, MMP1, and MMP3 transcription in WT infection compared to Δ*vhs* infection was considerably greater (8- to 15-fold), thereby confirming their *vhs*-dependent transcriptional downregulation.

Functional enrichment analysis of *vhs*-dependently downregulated genes again showed an enrichment for genes downregulated upon type II interferon exposure. Strikingly, however, we also observed a strong functional enrichment for several GO terms (adj. *P* ≤ 0.001; see Data Set S5), in particular “extracellular matrix (ECM) organization” (>32-fold enriched, adj. *P* < 10^–25^). This included fibronectin (FN1), integrin beta 1 (ITGB1), a subunit of integrin complexes binding fibronectin, and several genes encoding collagen alpha chains. Enrichment was also observed for “focal adhesion,” i.e., the integrin-containing, multiprotein complexes that anchor the cell to the ECM and connect it to the actin cytoskeleton (31, 32). Of note, the additional *vhs*-dependent genes, which we identified in the extended genome-wide analysis, were also significantly enriched for focal adhesion and ECM organization (>11-fold enriched, adj. *P* < 10^–23^).

**FIG 4:**
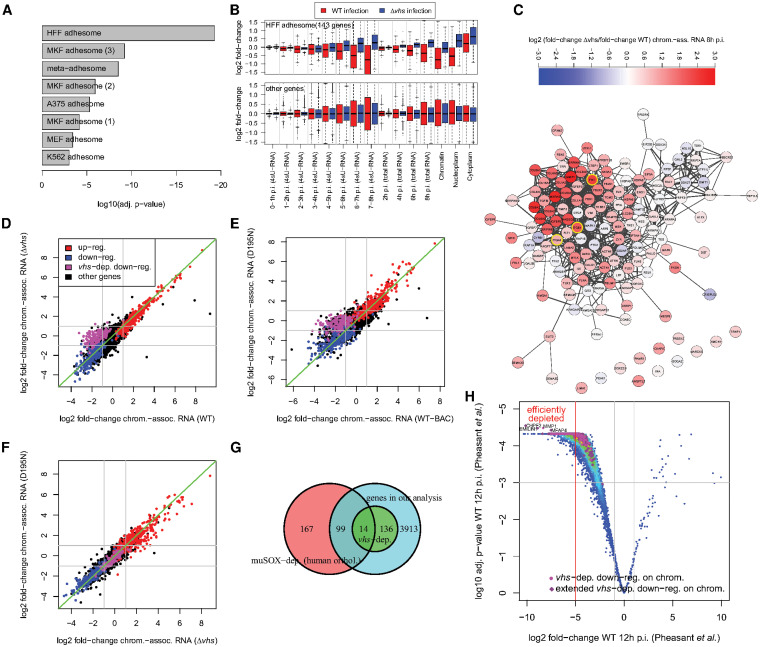
*vhs*-dependent transcriptional downregulation of the ECM and integrin adhesome. (A) *vhs*-dependently transcriptionally downregulated genes are significantly enriched for integrin adhesome components identified in six proteomics studies in HFF, MKF (three studies), A375, MEF, and K562 cells (33–38), and the meta-adhesome data were compiled by Horton et al. (33). A bar plot shows the log_10_ for multiple testing corrected *P* values from a Fisher exact test. (B) Boxplots showing the distribution of log_2_-fold change in 4sU-RNA, total RNA, and subcellular RNA fractions in WT (red) and Δ*vhs* (blue) infections for components of the integrin adhesome identified in HFF (36) (top panel) and all other genes (bottom panel). This shows a clear shift between WT and Δ*vhs* infections for the HFF integrin adhesome but not the remaining genes. (C) Protein-protein associations from the STRING database (39) for the HFF integrin adhesome. Colors indicate the log_2_ ratio between fold changes in Δ*vhs* and WT infections (see the color bar at the top). Red indicates less downregulation or more upregulation in Δ*vhs* infection than in WT infection, and blue indicates the opposite. Yellow borders highlight FN1, the canonical ligand of integrin adhesion complexes, and integrin subunits. The network was visualized with Cytoscape (82). (D to F) Comparison of log_2_-fold changes in chromatin-associated RNA for the repeat experiment of WT and Δ*vhs* infections at 8 h p.i. (D) and infection with the D195N mutant and its parental BAC-derived virus (WT-BAC) at 8 h p.i. (E), as well as D195N and Δ*vhs* infections (F). Colors indicate the regulation in chromatin-associated RNA in our original experiment (see Fig. 3C). Red = upregulated in both WT and Δ*vhs* infection, blue = downregulated in both, magenta = downregulated in a Δ*vhs* dependent manner, black = not regulated. (G) Venn diagram comparing human orthologues of muSOX-dependent genes identified by Abernathy et al. (18) against *vhs*-dependent genes identified in our study. The overlap of muSOX-dependent genes to the genes included in our analysis is also shown. A Fisher exact test was performed on the numbers in the light and dark cyan and green fields (*P* = 4.27 × 10^–5^). (H) Volcano plot showing the log_2_-fold change in total RNA in HSV-1 infection at 12 h p.i. and multiple testing adjusted *P* values from the study of Pheasant et al. (19). The original and extended set of *vhs*-dependently transcriptionally downregulated genes from our study are marked in magenta and violet, respectively. Genes defined as efficiently depleted by *vhs* by Pheasant et al. (log_2_-fold change < −5) are left of the red vertical line. *vhs*-dependent genes are among the most significantly downregulated genes. Gene symbols are shown for the four genes with lowest adjusted *P* values.

Composition of integrin adhesion complexes after induction by their canonical ligand FN1 has been determined by several quantitative proteomics studies in mouse and human cells, including HFF (33–38). Horton et al. consolidated these data into a meta-adhesome of 2,412 proteins found in at least one of six high-quality studies (33). Adhesome components identified in the individual proteomics studies, as well as the meta-adhesome, were significantly enriched among genes downregulated in a *vhs*-dependent manner (Fig. 4A, adj. *P* ≤ 0.001). The highest enrichment was found for the integrin adhesome components identified in HFF (>10-fold enrichment, adj. *P* = 5.3 × 10^–20^). Furthermore, genes of the HFF adhesome (143 genes included in our analysis) showed a systematic shift in regulation between WT and Δ*vhs* infections in total RNA, 4sU-RNA, and all RNA fractions (Fig. 4B). HFF adhesome components tended not to be (or at least less) transcriptionally downregulated in Δ*vhs* infection compared to WT infection, while the remaining genes showed no systematic shift. This shift was already visible from 4–5 h onward in 4sU-RNA and total RNA and when comparing later time points of Δ*vhs* infection to earlier time points of WT infection. Thus, *vhs*-dependent transcriptional downregulation is not an artifact of comparing different progression stages in the WT and *vhs* mutant life cycles in 8-h-p.i. chromatin-associated RNA. When inspecting the protein-protein association network for the HFF adhesome (from the STRING database [39]), the strongest differences between Δ*vhs* infection and WT infection were observed in the subnetwork around FN1 and integrin subunits (Fig. 4C).

To investigate whether *vhs*-dependent downregulation required *vhs* endonuclease activity, we performed RNA-seq of chromatin-associated RNA at 8 h p.i. using a *vhs* single-amino acid mutant (D195N) that no longer exhibited the mRNA decay activity but still binds to the translation initiation factors eIF4H and eIF4B (7). For comparison, we also included the parental BAC-derived WT virus (WT-BAC) as well as mock, WT and Δ*vhs* infection at 8 h p.i. (see Materials and Methods). This confirmed *vhs*-dependent transcriptional regulation in an independent experiment (Fig. 4D) and demonstrated that it requires *vhs* nuclease activity (Fig. 4E) since fold changes in D195N infection were extremely well correlated with Δ*vhs* infection (Fig. 4F). Of note, investigation of RNA-seq read alignments for genomic differences showed that the D195N point mutation was the only genome difference of the D195N mutant virus compared to WT-BAC. This confirms that the D195N mutant expresses the nuclease-null variant of *vhs*, rather than inadvertently no *vhs*. This analysis also confirmed the presence of the inactivating LacZ insertion in the Δ*vhs* mutant (22). We conclude that components of the integrin adhesome and ECM are transcriptionally downregulated during lytic HSV-1 infection by a *vhs*-nuclease-activity-dependent mechanism.

Gammaherpesviruses also encode an mRNA-targeting RNase, SOX, which is not homologous to *vhs*. Abernathy et al. recently showed that extensive mRNA cleavage by the murine gammaherpesvirus 68 (MHV68) endoribonuclease muSOX and subsequent Xrn1-mediated mRNA degradation leads to transcriptional repression for numerous genes (18). The same phenomenon was observed for several genes by qRT-PCR if the HSV-1 *vhs* protein was exogenously expressed for 24 h. Abernathy et al. used 4sU-seq of WT MHV68 infection and infection with a muSOX-inactivating MHV68 mutant (ΔHS) and identified 342 muSOX-dependent genes. Although they found no clear links to specific biological processes in their functional enrichment analysis, the KEGG pathway “focal adhesion” was significantly enriched among muSOX-dependent genes (19 of 342 genes). To investigate whether *vhs*-dependent transcriptional downregulation of the integrin adhesome and ECM components might be mediated by general RNA degradation or represent a *vhs*-specific response, we analyzed the overlap of muSOX-dependent genes to our list of *vhs*-dependent genes (Fig. 4G). Only 14 of the 150 (9.3%) *vhs*-dependent genes were orthologues to muSOX-dependent genes. While this overlap was statistically significant (Fisher exact test, *P* = 4.27 × 10^–5^), it is nevertheless small. Although this provides some evidence that *vhs*-dependent transcriptional downregulation for most genes is distinct from general mRNA-decay-dependent transcriptional repression, more work is required to rule out effects of different cell, species or virus backgrounds.

A different explanation for the concerted downregulation of a set of functionally related genes could be *vhs*-mediated RNA degradation of a key cellular transcriptional regulator. We thus performed a motif search in promoters of *vhs*-dependently downregulated genes but surprisingly found no significantly enriched known or novel transcription factor binding motifs in the proximal promoter regions (bp −2,000 to +2,000 relative to the transcription start site). To recover more distal regulation, we also performed a motif search in open chromatin peaks from ATAC-seq (assay for transposase-accessible chromatin using sequencing) data in uninfected cells (21) within 10, 25, or 50 kb of *vhs*-dependently downregulated genes. While this recovered several motif hits for the AP-1 transcription factor, no significant enrichment compared to all identified open chromatin peaks was observed. Interestingly, however, the first *vhs*-dependent gene significantly downregulated in 4sU-RNA of WT infection at 2–3 h p.i. was the ETS transcription factor ELK3, one of three ternary complex factors (TCFs) that act as cofactors of serum response factor (SRF) (40). SRF has been shown to be vital for focal adhesion assembly in embryonic stem cells (41). TCF-dependent genes identified from simultaneous knockouts of all three TCFs, as well as SRF targets from ChIP-seq, have previously been determined in mouse embryonic fibroblasts (MEFs) (42). Though we found no significant enrichment for TCF-dependent genes or TCF-dependent SRF targets, SRF targets in general were significantly enriched (∼2.25-fold) among *vhs*-dependent genes (*P* = 3.9 × 10^–5^). Nevertheless, only 42 (28%) of *vhs*-dependent genes were SRF targets and 93% of SRF targets were not *vhs* dependent in our study; thus, other regulatory mechanisms have to be involved. Further work is required to clarify this issue.

Pheasant et al. observed large differences regarding the extent of *vhs*-induced loss in total RNA levels between different cellular genes at 12 h post-WT infection (19). Using qRT-PCR, these researchers showed that this reduction was *vhs* dependent based on two sets of genes that exhibited either high (COL6A2, MMP3, and MMP1) or low (GAPDH, ACTB, and RPLP0) reduction in total RNA levels in WT infection. Since actinomycin D treatment showed a similar stability of corresponding mRNAs in uninfected cells, these authors concluded that these differences were due to differences in the susceptibility of the respective transcripts to *vhs* cleavage activity. By RNA-seq and PCR on chromatin-associated rather than total cellular RNA, we demonstrated that all three of their PCR-confirmed highly *vhs*-sensitive genes are actually transcriptionally downregulated in a *vhs*-dependent manner. Moreover, genes defined as efficiently depleted during WT infection by Pheasant et al. (log_2_-fold change in total RNA at 12 h post-WT infection < –5) were significantly enriched for ECM organization (>3-fold, adj. *P* = 7.4 × 10^–7^). We thus hypothesized that a significant fraction of highly *vhs*-sensitive genes identified by Pheasant et al. might actually be *vhs*-dependently transcriptionally downregulated. Indeed, both original and additional *vhs*-dependently transcriptionally regulated genes identified in our genome-wide analysis were strongly enriched among efficiently depleted genes determined by Pheasant et al. (4.2- to 6.8-fold enrichment, *P* < 10^–27^) and were among the most significantly downregulated genes in total RNA at 12 h p.i. in WT infections (Fig. 4H). We conclude that *vhs*-dependent transcriptional downregulation notably contributes to reduced total mRNA levels of the respective genes later on in WT HSV-1 infection and thereby explains the previously observed strong *vhs*-dependent reduction of their mRNA levels.

### A common core of upregulated genes in WT and Δ*vhs* infection.

**FIG 5:**
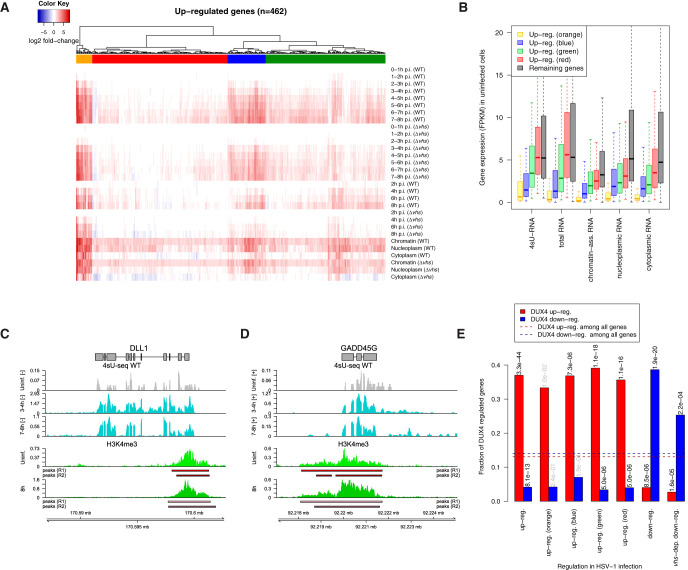
*vhs*-independent transcriptional upregulation of lowly expressed genes. (A) Heatmap of log_2_-fold changes in 4sU-RNA, total RNA, and subcellular RNA fractions for genes upregulated in both WT and Δ*vhs* infections (red in Fig. 3C). Genes were clustered according to Euclidean distances and Ward’s clustering criterion (see Materials and Methods). Four clusters were obtained at a distance threshold of 30 and are indicated by colored bars (orange, blue, green, and red). (B) Boxplots of the distribution of expression values (FPKM) in uninfected cells from 4sU-RNA, total RNA, and subcellular RNA fractions show low or no expression of strongly upregulated genes (orange cluster) in uninfected cells compared to other upregulated clusters (blue, green, and red) and remaining genes. (C and D) Strongly upregulated genes with low expression in uninfected cells, such as DLL1 (C, negative strand) and GADD45G (D, positive strand), are already primed for upregulation by H3K4me3 marks at their promoters. Tracks show read coverage (normalized to total number of mapped human reads; averaged between replicates) in uninfected and WT 4sU-RNA for selected time points (gray and cyan, top three tracks) and H3K4me3 ChIPmentation in uninfected cells and at 8 h after WT infection (green, bottom two tracks). Peaks identified in each replicate are shown separately below the H3K4me3 read coverage tracks. Gene annotation is indicated at the top. Boxes represent exons and lines introns. Genomic coordinates are shown on the bottom. For 4sU-seq data, only read coverage on the same strand as the gene is shown (+ = positive strand, – = negative strand). H3K4me3 ChIPmentation is not strand specific. (E) Bar plots showing the fraction of transcriptionally regulated genes in HSV-1 infection that are either up (red)- or down (blue)-regulated by doxycycline-inducible DUX4 (51). The results are shown separately for genes upregulated in both WT and Δ*vhs* infections, the four clusters of upregulated genes (the indicated colors refer to the cluster colors in Fig. 5A), and genes downregulated in both WT and Δ*vhs* infection, as well as genes downregulated in a *vhs*-dependent manner in WT infection. Horizontal dashed lines indicate the fraction of all analyzed genes regulated by DUX4. The numbers at the top of the bars indicate *P* values (corrected for multiple testing) for a Fisher exact test comparing the fraction of DUX4 up- or downregulated genes between each group of HSV-1 regulated genes to the background of all genes (black, adj. *P* ≤ 0.001; gray, not significant).

Analysis of chromatin-associated RNA identified a set of 462 genes that were significantly upregulated in both WT and Δ*vhs* infections (log_2_-fold change ≥1, adj. *P* ≤0.001, marked red in Fig. 3C). Only three genes were upregulated in WT but not or 2-fold less in Δ*vhs* infection. Thus, transcriptional upregulation during HSV-1 infection is independent of *vhs*. Clustering analysis of *vhs*-independent upregulated genes identified four subgroups that were distinguished mostly by how strongly and early in infection they were upregulated (Fig. 5A; see also Data Set S6). In particular, a set of 24 genes (marked orange in Fig. 5A) was upregulated both very early and strongly in WT and Δ*vhs* infections, with upregulation of 21 of these genes (91.7%) detectable in total RNA at 6 h p.i. or earlier in both WT and Δ*vhs* infections. Not surprisingly, several of these genes (e.g., RASD1, NEFM, and NPTX2) have previously been identified as highly upregulated in HSV-1 infection by microarray analysis on total RNA (43, 44), and 10 were significantly upregulated in total RNA at 12 h after WT infection in the Pheasant et al. data (19). Upregulation of all genes in the orange and blue clusters was also confirmed in 4sU-RNA. No enrichment for GO terms was observed either for individual clusters or all upregulated genes; however, the green and orange cluster were enriched for interferon type I-upregulated genes (adj. *P* = 1.68 × 10^–5^ and adj. *P* = 0.0019 for the green and orange clusters, respectively). Notably, 50% of genes in the orange cluster were upregulated by type I interferons (>4.5-fold enrichment).

One characteristic feature of upregulated genes in general and the orange cluster in particular was their low level of gene expression in uninfected cells (Fig. 5B). Notably, 71% of genes in the orange cluster were not or only very lowly expressed (fragments per kilobase of exon model per million mapped fragments [FPKM] in total RNA ≤ 1) in uninfected cells compared to 8% of all genes (Fisher exact test, *P* < 10^–13^). In total, 76 (17, 22, 32, and 5 from the orange, blue, green, and red clusters, respectively) upregulated genes (16.5%) were poorly expressed in uninfected cells. HSV-1-induced upregulation of genes not normally expressed has previously been reported for human alpha-globin genes (HBA1 and HBA2), which are normally only expressed in erythroid cells (45). RNA-seq analysis of these two duplicated genes is complicated by their high sequence similarity (>99% on coding sequence, 5′ untranslated regions and upstream of promoter [46]), since most reads can be mapped equally well to both genes and their promoter regions. Nevertheless, our data clearly confirmed that at least one of the two alpha-globin genes is transcribed during HSV-1 infection as early as 2–3 h p.i. and translated into protein at least from 4 h p.i. (according to our previously published Ribo-seq data [20]). Our analysis suggests that similar upregulation from no or low expression is observed for a number of other cellular genes. Since we used relatively strict criteria to exclude genes that only appeared to be expressed during infection due to read-in transcription, we also investigated more lenient criteria to identify the extent of induction for genes that are not expressed prior to infection (see Materials and Methods for details). These criteria applied to 17 of the upregulated genes (e.g., DLL1) and an additional 33 genes not included in our previous analysis. Manual inspection of these 33 genes confirmed transcriptional upregulation for only 13 genes (IRF4, RRAD, FOSB, ARC, CA2, DIO3, DLX3, GBX2, ICOSLG, MAFA, MAFB, NGFR, and PCDH19). Of these, 6 and 8 were upregulated by type I and II interferons, respectively. In summary, only a small fraction of genes not expressed in uninfected fibroblasts is induced by HSV-1 infection.

To start investigating how the rapid upregulation of these genes might be achieved, we performed ChIPmentation (47) of H3K4me3 histone marks (two replicates each in uninfected cells and at 8 h after WT infection). H3K4me3 has been reported to regulate assembly of the preinitiation complex for rapid gene activation (48). Furthermore, a bivalent chromatin modification pattern combining H3K4me3 and H3K27me3 has been described in embryonic stem (ES) cells, which serves to keep silenced developmental genes poised for activation (49). Across all four samples, we identified 32,601 unique nonoverlapping peak regions, which were strongly enriched around gene promoters (see Data Set S7). In total, 98.7% of analyzed genes exhibited H3K4me3 peaks around the promoter in both replicates of uninfected cells. Notably, this also applied to 21 of the 24 genes in the orange cluster (87.5%; see Fig. 5C and D for examples). Only NPTX1 and NPTX2 showed no significant H3K4me3 promoter peak in either replicate of uninfected cells, but both showed peaks in at least one replicate of the infected cells. In total, 97.8% of all upregulated genes and 92.1% of upregulated genes that were not or lowly expressed in uninfected cells (total RNA FPKM ≤1) showed significant peaks in both replicates of uninfected cells. In summary, this indicates strong, early, *vhs*-independent transcriptional upregulation of a small number of poorly expressed genes which are already poised for expression by H3K4me3 marks at their promoters.

Recently, Full et al. reported that the germ line transcription factor DUX4 (double homeobox 4) and several of its targets are highly upregulated by HSV-1 infection (50). We thus compared genes up- or downregulated by doxycycline-inducible DUX4 (51) with genes transcriptionally regulated in HSV-1 infection (Fig. 5E). We found that HSV-1 upregulated genes were significantly (Fisher exact test, adj. *P* ≤0.001) enriched for DUX4 upregulation, and HSV-1 downregulated genes were significantly enriched for DUX4 downregulation. Notably, the fraction of genes upregulated by DUX4 was similar (∼36%) for all clusters of HSV-1 upregulated genes, independent of their expression in uninfected cells. Interestingly, however, genes that were transcriptionally downregulated in HSV-1 infection in a *vhs*-mediated manner were less enriched for DUX4-mediated downregulation than genes for which transcriptional downregulation was independent of *vhs.* Moreover, enrichment for adhesome components was more pronounced among *vhs*-dependent genes not downregulated by DUX4 than among those downregulated by DUX4. Thus, while DUX4 is a major transcriptional regulator in HSV-1 infection, it is not responsible for *vhs*-mediated downregulation of the integrin adhesome.

### *vhs*-dependent transcriptional downregulation impacts on cellular protein levels.

**FIG 6:**
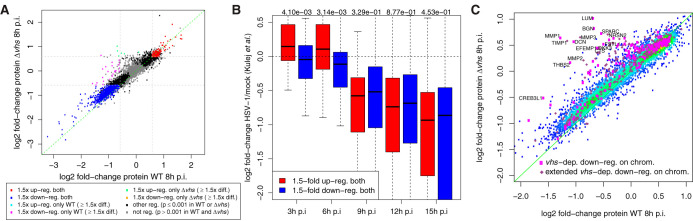
Impact of HSV-1 infection on protein levels. (A) Comparison of log_2_-fold change in protein levels at 8 h p.i. between WT (*x* axis) and Δ*vhs* (*y* axis) infections. Up- or downregulated proteins (≥1.5-fold change, adj. *P* ≤ 0.001) in both WT and Δ*vhs* infections are indicated in red and blue, respectively. Proteins downregulated in a *vhs*-dependent manner (≥1.5-fold downregulated, adj. *P* ≤ 0.001 in WT infection; <1.5-fold downregulated in Δ*vhs* infection, as well as a >1.5-fold difference in regulation) are marked in magenta. Green indicates proteins that are upregulated in Δ*vhs* infection but not in WT infection, with a >1.5-fold difference in fold changes. (B) Boxplots of log_2_-fold change of normalized protein iBAQ intensities from Kulej et al. (53) for HSV-1 infection versus mock infection for proteins that are either significantly upregulated (red in panel A) or downregulated (blue in panel A) in our study and significantly regulated between any pair of time points in the Kulej et al. time course (ANOVA, *P* < 0.001). *P* values for the Wilcoxon rank sum test comparing log_2_-fold changes for the respective time points from the Kulej et al. study between our up- and downregulated proteins are indicated at the top. (C) Comparison of protein log_2_-fold changes for *vhs*-dependently transcriptionally regulated genes (marked magenta and violet as in Fig. 4H) and other genes (color-coded according to density of points: from red = high density to blue = low density). Gene symbols are shown for genes with a ≥2-fold increase in protein fold changes in Δ*vhs* infection compared to WT infection.

To investigate how changes in total RNA levels and transcription alter protein levels in infected cells, we performed a tandem mass tag (TMT)-based quantitative proteomic analysis of WT- and Δ*vhs*-infected HFF at 0 and 8 h p.i. (*n* = 3 replicates). In total, 7,943 proteins were identified (see Data Set S8). No filtering based on read-in transcription was performed, since read-through transcripts are neither exported nor translated (20, 21). Protein fold changes were poorly correlated with fold changes in total RNA, 4sU-RNA, or subcellular RNA fractions (*r_S_* ≤0.21) and generally tended to be less pronounced. Both observations are consistent with the higher stability of proteins compared to mRNAs (∼5 times more stable in mouse fibroblasts [52]); thus, changes in *de novo* transcription and total RNA levels commonly take >8 h to significantly impact protein levels. Consequently, protein fold changes were very well correlated between WT and Δ*vhs* infection (Fig. 6A, *r_S_* = 0.96), and only a few cellular proteins showed a significant difference between WT and Δ*vhs* infection. Due to the less pronounced changes, we determined differentially expressed proteins with a >1.5-fold change (adj. *P* ≤ 0.001, Fig. 6A). Most differentially expressed proteins were concordantly regulated either down (1,444 genes, 73%) or up (499 genes, 25.3%) in both WT and Δ*vhs* infections. It should be noted that, similar to RNA-seq data, protein fold changes only represent relative changes in the presence of a global loss in cellular protein levels. Thus, some upregulated proteins may simply be less/not downregulated compared to most other proteins.

To evaluate consistency with previously identified HSV-1-regulated proteins, we compared our concordantly up- or downregulated proteins against significantly regulated proteins (analysis of variance [ANOVA], *P* ≤ 0.001) from the proteomics time course for 3, 6, 9, and 12 h p.i. HSV-1 infection from the study of Kulej et al. (53). This included 4,613 proteins and 53% of the proteins from our study. While only 33 of the upregulated and 109 of the downregulated proteins from our study were also significantly regulated in the Kulej et al. study, the direction of regulation was consistent for both sets of genes until 6 h p.i. (Fig. 6B). From 9 h p.i., both up- and downregulated proteins from our study that were significantly regulated in the study of Kulej et al. tended to be downregulated to a similar extent. The relatively small overlap between our study and the study by Kulej et al. is not surprising considering the generally low overlap of previous proteomics studies on HSV-1 infection (53–55). Most likely, this is explained by differences between the cells used and the infection doses, resulting in different infection kinetics.

Concordantly downregulated proteins were significantly (adj. *P* ≤ 0.001; see Data Set S9) enriched for a number of GO terms, including “nucleotide-sugar biosynthetic process” (>77-fold enriched), “canonical glycolysis” (>15-fold), “viral budding” (>9-fold), and “activation of MAPK activity” (>4-fold). Interestingly, meta-adhesome (but not HFF adhesome) components were also significantly enriched (>1.9-fold), indicating that concordantly downregulated proteins interact with the core adhesome, rather than are a part of it. Interestingly, concordantly upregulated proteins were highly enriched for mitochondrial proteins (>7-fold, 186 proteins) but significantly depleted of meta-adhesome components. To test how many of these proteins were upregulated following a significant increase in their total RNA levels, we determined genes that were significantly upregulated in total RNA in both WT and *Δvhs* infections (19 genes). Five of these were also upregulated at protein level, including four genes that were upregulated in chromatin-associated RNA (RASD1, SNAI1, CBX4, and ITPR1). Thus, transcriptional upregulation can have a small but measurable effect on protein levels by 8 h p.i. Only a few genes showed a significant differential effect (24 downregulated in WT only, 6 upregulated in Δ*vhs* only). Strikingly, the 24 proteins downregulated in a *vhs*-dependent manner were strongly enriched for HFF adhesome components (>12-fold) and ECM organization (>23-fold). Accordingly, 9 of 14 (64%) proteins downregulated only in WT infection and included in our RNA-seq analysis were downregulated in chromatin-associated RNA in a *vhs*-dependent manner. An analysis of protein fold changes for all *vhs*-dependently transcriptionally downregulated genes (including our extended set) demonstrated that a significant number of respective proteins were either less downregulated or (relatively) more upregulated in Δ*vhs* infection than in WT infection (Fig. 6C). Many of these were components of the integrin adhesome or were involved in ECM organization. Thus, *vhs*-dependent transcriptional downregulation impacts protein levels of the respective genes already by 8 h p.i.

## DISCUSSION

HSV-1 infection drastically alters host RNA metabolism at all levels by impairing host mRNA synthesis, processing, export and stability. Here, we differentiate and quantify their individual contributions to the RNA expression profile by combining RNA-seq of total, newly transcribed (4sU-RNA) and subcellular RNA fractions in WT and Δ*vhs* infection. While it is important to note that the WT and Δ*vhs* time course experiments were performed independently, we carefully standardized the experimental conditions, e.g., by infecting the same batch of cells following the same number of splits after thawing, as well as using the same batch of fetal bovine serum (FBS), to achieve a maximum level of reproducibility. Indeed, all major results were confirmed in experiments which were performed in parallel for WT and Δ*vhs* infections.

We developed a mathematical model to quantify both the loss of transcriptional activity and the changes in *vhs* nuclease activity based on the correlations between RNA half-lives and total RNA fold changes during the first 8 h of infection. This showed a drop in transcriptional activity down to 10 to 20% of the original level by 8 h p.i. in Δ*vhs* infection, consistent with the well-described general loss of Pol II from host chromatin (14, 15). The WT HSV-1 time course depicted a rapid increase in *vhs*-dependent degradation, with 20 to 30% of all cellular mRNAs degraded per hour by 2 h p.i., consistent with the well-described role of *vhs* upon viral entry. Although *vhs* activity did not further rise from 4 h p.i. despite increasing *vhs* protein levels, it was constantly maintained until 8 h p.i. The kinetics of the viral life cycle are incorporated in our ODE model via the functions describing *vhs* activity and cellular transcriptional activity. Since *vhs* activity and cellular transcriptional activity cannot be estimated simultaneously in WT infection, we used the estimated changes of host transcriptional activity from Δ*vhs* infection in WT infection. However, no data from the Δ*vhs* time course was used in the modeling of WT infection, only the estimated function describing the kinetics of host transcriptional activity. Considering the slower progression of Δ*vhs* infection, we may thus have underestimated the drop in transcriptional activity in WT infection. However, if transcriptional activity drops even faster and further in WT infection, *vhs* activity would have to increase even faster and to higher levels to explain the observed negative correlations between RNA half-lives and total RNA fold changes in WT infection. It is important to note that our findings do not contradict previous reports on the posttranscriptional downregulation of *vhs* activity by its interaction with VP16 and VP22 (9–11). As previously noted (9), counter-regulation of *vhs* activity is not complete, but VP16 and VP22 clearly serve to prevent a further detrimental increase in *vhs* activity during infection. Their activity thus explains the plateau we observed for *vhs* activity despite substantially increasing *vhs* protein levels. Moreover, application of our model to total RNA fold changes at 12 h p.i. WT infection from the study of Pheasant et al. confirmed deactivation of *vhs* between 8 and 12 h p.i.

Pheasant et al. also noted that *vhs*-dependent reduction in total RNA levels varied widely between genes at 12 h p.i. and hypothesized that this might indicate differences in susceptibility to *vhs*-mediated degradation between transcripts (19). Furthermore, these researchers excluded an influence of basal transcription rates and RNA half-lives for the three genes whose high *vhs* sensitivity they confirmed by PCR. However, we here show that all three genes they selected for experimental validation are actually transcriptionally downregulated in a *vhs*-dependent manner. Together with the effects of *vhs* on RNA stability, this translates into a significant reduction in the corresponding protein levels by 8 h p.i. Accordingly, results from these three genes cannot be extrapolated to genes downregulated in total RNA *only* through *vhs*-mediated RNA decay. Instead, our ODE model suggests that gene-specific differences in mRNA half-lives substantially shape the variability in total mRNA changes between genes at least until 8 h p.i. This does not exclude a contribution of other factors, e.g., *vhs*-induced nuclear retention of cellular mRNAs shown by Pheasant et al. (19) or differences in translation rates between different mRNAs (and thus translation-initiation-dependent mRNA cleavage by the *vhs* protein), which we did not consider in our model. In particular, *vhs*-dependent transcriptional downregulation contributes substantially to the reduction in total RNA levels for the respective genes. Furthermore, a recent study identified a set of 74 genes that escape degradation by four herpesviral endonucleases, including *vhs* (56). Almost all of these genes were excluded from our analysis due to low expression (87%), read-in transcription (7%), or proximity to nearby genes (3%). Two genes, however, which were not excluded, (C19orf66 and ARMC10) indeed did not show any significant change in any of our data. Selective targeting of *vhs* to unstable mRNAs via AU-rich elements in a translation-independent manner has also been reported (57). We thus do not exclude that some transcripts are more or less susceptible to *vhs*-mediated decay than others. However, we conclude that strong *vhs*-dependent reductions in total mRNA levels are not necessarily a consequence of increased susceptibility of individual transcripts to *vhs*-mediated RNA cleavage.

In contrast to total cellular RNA changes, fold changes in newly transcribed and, in particular, chromatin-associated RNA were surprisingly similar between WT and Δ*vhs* infections. This enabled us to decipher gene-specific transcriptional regulation that is either dependent or independent of *vhs*. Although we performed the combined total RNA-seq and 4sU-seq time courses for both viruses in two separate experiments, the high correlation of the 4sU-RNA fold changes confirmed that it was valid to also compare the corresponding total RNA-seq time course data.

While the analysis of chromatin-associated RNA eliminated the bias originating from *vhs* activity and the global loss in transcription, read-in transcription leading to seeming, but nonfunctional, induction of genes has to be taken into account in all gene expression profiling studies independent of the type of profiled RNA. By excluding genes with evidence of read-in transcription from our analysis, we ascertained that all identified induced genes represent true upregulation and not artifacts from read-in transcription. Notably, although most strongly upregulated genes identified in our study have been reported in previous studies on HSV-1-induced differential host expression (e.g., RASD1 [19, 43, 58, 59]), several previously reported genes, which were thought to be induced by HSV-1, are actually only seemingly induced due to read-in transcription, e.g., ZSCAN4 (43, 60), SHH (58), and FAM71A (19).

Around 30% of all upregulated genes and 50% of the most strongly upregulated genes (orange cluster) were upregulated by type I interferons (IFNs). Moreover, DUX4 was confirmed as a major transcriptional regulator in both WT and Δ*vhs* infections for both up- and downregulated genes (37% of upregulated genes were previously found to be upregulated by DUX4 and 39% of downregulated genes were downregulated by DUX4, Fig. 5E). Although there was some overlap between DUX4 and IFN-induced genes among the HSV-1-induced genes, it was not significantly larger than expected at random. Interestingly, the DUX4 upregulated gene TRIM43 was recently identified as a herpesvirus-specific antiviral factor independent of the type I IFN response (50). This suggests that DUX4-mediated regulation in HSV-1 infection may represent an alternative pathway which augments the host intrinsic immune response.

A key finding of our study is the *vhs*-dependent, transcriptional downregulation of proteins involved in the integrin adhesome and ECM organization, which required *vhs* nuclease activity. Suppression of ECM protein synthesis during HSV-1 infection has already been reported over 30 years ago for the canonical integrin ligand FN1, type IV procollagen, and thrombospondin (61). Recently, this was confirmed for a few other ECM components in human nucleus pulposus cells in both lytic and latent HSV-1 infections (62). A *vhs* dependency of downregulation was previously reported for FN1 (63) but was ascribed to the effect of *vhs* on FN1 RNA stability. This further highlights the pitfalls in ascribing all *vhs*-dependent effects on total RNA levels solely to *vhs*-mediated RNA decay. In contrast, our data demonstrate that *vhs*-dependent downregulation of specific genes is augmented by *vhs*-dependent repression of transcription. Notably, although *vhs*-dependent downregulation of the ECM and adhesome can largely be confirmed in total RNA, it is challenging to distinguish it from *vhs*-mediated mRNA degradation. The transcriptional effects only become obvious when analyzing chromatin-associated RNA.

Interestingly, transcriptional downregulation of ECM and integrin adhesome genes was dependent on the nuclease activity of *vhs*. Recently, muSOX-mediated RNA decay was reported to trigger transcriptional repression at late times of lytic MHV68 infection (18). Although HSV-1 *vhs* activity also triggered this phenomenon within 24 h of expression, the cellular genes transcriptionally regulated in a *vhs*-dependent manner during the first 8 h of HSV-1 infection showed little overlap to the genes affected by the transcriptional effects of muSOX-induced RNA degradation. To date, the molecular mechanism underlying the transcriptional shutoff induced upon extensive cytoplasmic RNA degradation remains unclear. While we cannot fully exclude that *vhs*-dependent transcriptional downregulation of the integrin adhesome and ECM components marks the advent of this effect, our data are more likely to be explained by a distinct gene-specific function of *vhs* with more widespread transcriptional repression only becoming relevant at later times of infection.

An alternative explanation for the *vhs*-dependent repression of such a functionally connected cellular network of genes is that *vhs* nuclease activity results in a rapid depletion of transcripts of key, short-lived cellular transcription factor(s) governing these genes. It is unclear, however, why only a single or a very small number of transcription factors would suffer so much more dramatically from *vhs* nuclease effects. It is indeed surprising that *vhs*-mediated mRNA degradation does not cause a similarly pronounced dysregulation downstream of short-lived transcription factors involved in other processes. However, the surprisingly high correlation between fold changes in WT and Δ*vhs* infections observed in chromatin-associated RNA excludes gross global effects of mRNA degradation of cellular transcriptional factors. Furthermore, no enrichment of any known or novel transcription factor binding motif could be identified in both proximal promoter regions or more distal open chromatin regions identified by ATAC-seq. Promoter analysis applied to all expressed HFF adhesome genes identified only one significant motif which was only observed in <6% of genes, suggesting that there is no single key transcriptional regulator for the integrin adhesome. Nevertheless, *vhs* may still directly interact with or target a major cellular transcription factor that governs the expression of the integrin adhesome and ECM via distal enhancers. Notably, ELK3, a TCF cofactor of SRF, was downregulated in a *vhs*-dependent manner early on in infection. While TCF-dependent genes were not enriched among *vhs*-dependent genes, an ∼2-fold enrichment of SRF targets was observed. Since TCF-dependent genes were determined by triple knockouts of all three TCFs (42), not all TCF-dependent genes likely depend on ELK3. While our Western blot analysis of ELK3 protein abundance was inconclusive (data not shown), quantitative proteomics suggested at least a weak change (1.6-fold) between WT and Δ*vhs* infections. Thus, ELK3-dependent reduced recruitment of SRF may still play a role. Alternatively, posttranscriptional processes, which have been linked to transcriptional control of focal adhesions, may also be relevant for *vhs*-dependent downregulation. For instance, Rho signaling can result in nuclear translocation of the SRF cofactor MRTF-A and prevention of this translocation results in lower expression of cytoskeletal/focal adhesion proteins (64). Furthermore, upregulation of nuclear actins lead to transcriptional downregulation of a number of adhesion proteins (65), such as ITGB1 and MYL9, which were also downregulated in a *vhs*-dependent manner in HSV-1 infection.

Untangling the molecular mechanisms underlying specific *vhs*-mediated downregulation of the integrin adhesome and ECM will be difficult without knowledge of the responsible cellular transcription factor(s) and confounded by the pleotropic effects of *vhs* nuclease activity. Nevertheless, we could show that *vhs*-dependent transcriptional downregulation has a clear impact on protein levels already by 8 h p.i., as confirmed by quantitative whole-cell proteomics. Proteins with strong *vhs*-dependent reduction at 8 h p.i. include matrix metallopeptidases MMP1 to MMP3, which are involved in degradation of ECM proteins, their inhibitor TIMP1 as well as other MMP-upregulating or -interacting proteins (LUM, SPARC, and THBS2).

In summary, our analyses provide a comprehensive, quantitative picture of the molecular mechanisms that govern profound alterations in the host cell transcriptome and proteome during lytic HSV-1 infection.

## MATERIALS AND METHODS

### Cell culture and infections.

Human fetal foreskin fibroblasts (HFF) were purchased from ECACC (catalog no. 86031405) and cultured in Dulbecco modified Eagle medium with 10% FBS Mycoplex and 1% penicillin-streptomycin. HFF were utilized from passages 11 to 17 for all high-throughput experiments. This study was performed using WT HSV-1 strain 17 (data taken from previous studies [20, 21]) and its *vhs*-inactivated mutant (Δ*vhs*) (22). In this mutant, *vhs* was inactivated by inserting the *lacZ* coding sequence at codon 251. It thus produces a 250-residue amino-terminal *vhs* fragment and deletes residues 251 to 485. While the mutant may thus retain some unknown activity of *vhs*, its nuclease activity and thus all of its known functions are inactivated. Virus stocks were produced in baby hamster kidney (BHK) cells (obtained from the American Type Culture Collection) as described previously (20). HFF were infected with HSV-1 24 h after the last split for 15 min (for total RNA-seq, 4sU-seq, and RNA-seq of subcellular fractions) or 1 h (for RNA-seq of chromatin-associated RNA, including the *vhs* D195N mutant), at 37°C using an MOI of 10. Subsequently, the inoculum was removed, and fresh medium was applied to the cells.

The *vhs* D195N mutant virus was constructed via *en passant* mutagenesis (66). Mutagenesis templates were generated using the PCR primers GTATATCTGGCCCGTACATCGATCT and GGTCAGTGTCCGTGGTGTACACGTACGCGACCGTGTTGGTGTGATAGAGGTTGGCGCAGGCATTGTCCGCCTCCAGCTGACCCGAGTTAAAGGATGACGACGATAAGTAGGG to amplify the kanamycin resistance cassette flanked by Isce-I restriction sites from vector pEP-Kan. Additional homologies for recombination were added to this product by PCR using the primers GGTCAGTGTCCGTGGTGTAC and TTCTGTATTCGCGTTCTCCGGGCCCTGGGGTACGCCTACATTAACTCGGGTCAGCTGGAGGCGGACAATGCCTGCGCCAACCTCTATCACGTATATCTGGCCCGTACATCGATCT before electroporation into Escherichia coli strain GS1783 containing the pHSV(17+)Lox BAC (67). BAC DNA was purified using a NucleoBond BAC 100 kit (Macherey-Nagel, catalog no. 740579) and transfected for virus reconstitution into BHK-21 cells with Lipofectamine 3000 (Thermo Fisher, L3000-075).

### Preparation of RNA samples.

Sample preparation for 4sU-seq in Δ*vhs* infection was performed as reported previously for WT HSV-1 (20). In brief, 4-thiouridine (4sU) was added to the cell culture medium for 60 min at –1, 0, 1, 2, 3, 4, 5, 6, or 7 h p.i. (2 × 15-cm dishes per condition) during Δ*vhs* infection to a final concentration of 500 μM (*n* = 2 replicates). Subsequently, the medium was aspirated and the cells were lysed with TRIzol (Invitrogen). Total RNA and newly transcribed RNA fractions were isolated from the cells as described previously (28). In an independent experiment, subcellular RNA fractions (cytoplasmic, nucleoplasmic, and chromatin-associated RNA) in mock infections and 8 h p.i. of WT and Δ*vhs* infections were prepared as previously described (*n* = 2 replicates) (21). To assess the role of *vhs* nuclease activity in regulation of ECM and integrin adhesome genes, chromatin-associated RNA in mock, WT, Δ*vhs*, *vhs* D195N, and WT-BAC infections at 8 h p.i. (*n* = 2 replicates) was prepared.

### Library preparation and RNA sequencing.

Sequencing libraries were prepared using the TruSeq stranded total RNA kit (Illumina). rRNA depletion was performed after DNase treatment for total RNA and all subcellular RNA fractions using Ribo-zero but not 4sU-RNA samples. Sequencing of 75 bp paired-end reads was performed on a NextSeq 500 (Illumina) at the Core Unit Systemmedizin (Würzburg).

### Quantitative reverse transcription-PCR.

Chromatin-associated RNA was isolated in TRIzol as described above and then DNase treated and purified with the Direct-zol RNA Miniprep kit (Zymo, catalog no. R2051). cDNA was synthesized using the “optional procedure” of the BioScript All-in-One cDNA Synthesis SuperMix (Biotool, catalog no. B24403). Real-time PCR performed with SYBR green qPCR Master Mix (Bimake, catalog no. B21202) using the recommended three-step protocol and 1 μM concentrations of the following primer pairs for each gene: 18S rRNA (GCAATTATTCCCCATGAACG and GGGACTTAATCAACGCAAGC), ARF4 (CCTTCTGCTTCTGCCCATCA and CGCATCTGCTTCTTGCCAAA), CNBP (AAACTGGTCATGTAGCCATCAAC and AATTGTGCATTCCCGTGCAAG), COL6A2 (GCAACGACTACGCCACCAT and GACCTTGATGATGCGGTTGA), MMP1 (TAGTGGCCCAGTGGTTGAAA and GGGCTGCTTCATCACCTTCA), and MMP3 (AGTCTCTGTGAATTGAAATGTTCG and AGTTCCCTTGAGTGTGACTCG). cDNA samples were diluted 1:10 in water for protein-coding genes and 1:1,000 for 18S rRNA; they were then diluted 1:4 for the final reaction, which was performed on a LightCycler 96 (Roche). *C_q_* values for each gene were calibrated to 18S rRNA for that sample, and relative expression between samples was calculated using the ΔΔ*C_q_* method.

### H3K4me3 ChIPmentation.

The full description of H3K4me3 ChIPmentation is included in Text S10 in the supplemental material.

### Preparation of samples for proteomic analysis.

HFF were infected with WT or Δ*vhs* HSV-1 for 8 h at an MOI of 10. Infections were conducted in triplicate, with four uninfected controls (10 samples in total). Washed cells were snap-frozen in liquid nitrogen. Cells were lysed in by resuspending in 100 μl 2% sodium dodecyl sulfate (SDS) and 50 mM tetraethylammonium bromide (TEAB; pH 8.5), followed by a 10-min (30-s on/off duty cycle) sonication in a Bioruptor sonicator (Diagenode). Lysates were quantified by a BCA assay, and 50-μg portions of each sample were reduced and alkylated with 10 mM Tris(2-carboxyethyl)phosphine (TCEP) and 40 mM iodoacetamide for 20 min at room temperature in the dark. Samples were diluted to 500 μl with 8 M urea–50 mM TEAB, assessed using 30-kDa Vivacon centrifugal ultrafiltration devices (Sartorius), and concentrated according to the manufacturer’s instructions. Samples were resuspended and concentrated in 8 M urea a further three times to remove residual SDS. There were a further three washes with digestion buffer (0.5% sodium deoxycholate [SDC], 50 mM TEAB) before the samples were resuspended in approximately 50 μl of digestion buffer with 1 μg of trypsin (proteomics grade; Thermo Fisher). Filter units were then incubated in at 37°C overnight in a box partially filled with water to reduce evaporation. Peptides were recovered into a fresh tube by centrifugation and a further wash with 50 μl of digestion buffer. The SDC was removed from each sample by precipitation with the addition of formic acid and two-phase partitioning with ethyl acetate. Peptides were then dried under vacuum. For TMT labeling, the samples were resuspended in 42 μl of 100 mM TEAB, and then 0.4 mg of each TMT reagent in 18 μl of anhydrous acetonitrile was added, followed by vortexing to mix the sample and incubation at room temperature for 1 h. A small aliquot of each sample was analyzed by liquid chromatography-mass spectrometry (LC-MS) to confirm the labeling efficiency, and samples were pooled 1:1 according to the total TMT reporter intensity in these quality control runs. The pooled sample was then acidified and subjected to solid-phase extraction cleanup using 50-mg tC18 cartridges (Waters) before drying under a vacuum.

### Basic pH reversed-phase fractionation.

Samples were resuspended in 40 μl of 200 mM ammonium formate (pH 10) and transferred to a glass high-pressure liquid chromatography (HPLC) vial. BpH-RP fractionation was conducted on an Ultimate 3000 UHPLC system (Thermo Scientific) equipped with a Kinetex EVO column (2.1 mm by 15 cm, 1.7 μm; Phenomenex). Solvent A was 3% acetonitrile, solvent B was 100% acetonitrile, and solvent C was 200 mM ammonium formate (pH 10). Throughout the analysis, solvent C was kept at a constant 10%. The flow rate was 400 μl/min, and the UV absorbance was monitored at 280 nm. Samples were loaded in 90% solvent A for 10 min before a gradient elution of 0 to 10% solvent B over 10 min (curve 3), 10 to 34% solvent B over 21 min (curve 5), and 34 to 50% solvent B over 5 min (curve 5), followed by a 10-min wash with 90% solvent B. 15-s (100 μl) fractions were collected throughout the run. Fractions containing peptide (as determined by 280-nm light absorbance) were recombined across the gradient to preserve orthogonality with on-line low-pH RP separation. For example, fractions 1, 25, 49, 73, and 97 were combined, dried in a vacuum centrifuge, and stored at –20°C until LC-MS analysis.

### Mass spectrometry.

Samples were analyzed on an Orbitrap Fusion instrument on-line with an UltiMate 3000 RSLCnano UHPLC system (Thermo Fisher). Samples were resuspended in 10 μl of 5% dimethyl sulfoxide–1% trifluoroacetic acid (TFA), and 5 μl of each fraction was injected. The trapping solvent was 0.1% TFA, the analytical solvent A was 0.1% formic acid, and solvent B was acetonitrile with 0.1% formic acid. Samples were loaded onto a trapping column (300 μm × 5 mm PepMap cartridge trap; Thermo Fisher) at 10 μl/min for 5 min. Samples were then separated on a PepMap C_18_ column (50 cm × 75 μm i.d., 2-μm particle size; Thermo Fisher). The gradient was 3 to 10% solvent B over 10 min, 10 to 35% solvent B over 155 min, 35 to 45% solvent B over 9 min, followed by a wash with 95% solvent B for 5 min and reequilibration with 3% solvent B. Eluted peptides were introduced by electrospray to the MS by applying 2.1 kV to a stainless-steel emitter (5 cm × 30 μm; Thermo Fisher). During the gradient elution, MS1 spectra were acquired in the Orbitrap, and the collision-induced dissociation (CID)-MS2 was acquired in the ion trap. Synchronous precursor selection-isolated MS2 fragment ions were further fragmented using higher-energy collisional dissociation to liberate reporter ions which were acquired in the Orbitrap (MS3).

### Data processing.

Raw files were searched using Mascot (Matrix Science) from within Proteome Discoverer v2.1 (Thermo Fisher) against the UniProt human database with appended common contaminants and the UniProt HSV reference proteome. The peptide spectrum match false discovery rate (FDR) was controlled at 1% using Mascot Percolator. The reporter ion intensities of proteins with high (1%) and medium (5%) FDRs were taken and subjected to LIMMA *t* test in R. *P* values were adjusted for multiple testing by using the Benjamini-Hochberg method (68). Proteins with extremely high standard deviations between replicates in (>99 percentile) in either WT or Δ*vhs* infection were excluded from further analysis.

### Processing of next-generation sequencing data.

Sequencing reads were mapped against (i) the human genome (GRCh37/hg19), (ii) human rRNA sequences, and (iii) the HSV-1 genome (HSV-1 strain 17, GenBank accession code JN555585) using ContextMap v2.7.9 (69) (using BWA as short read aligner [70] and allowing a maximum indel size of three and at most five mismatches). For the two repeat regions in the HSV-1 genome, only one copy each was retained, excluding nucleotides 1 to 9,213 and 145,590 to 152,222. ContextMap produces unique mappings for each read; thus, no further filtering was performed. Read coverage was visualized using Gviz (71) after normalizing to the total number of mapped human reads and averaging between replicates. For identification of enriched H3K4me3 regions (=peaks), BAM files with mapped reads were converted to BED format using BEDTools (72) (v2.24.0), and peaks were determined from BED files using F-Seq with default parameters (73). Only peaks with lengths of ≥500 nucleotides were considered. Unique nonoverlapping peaks were identified by merging overlapping peaks across all samples using BEDTools. Overlaps of identified peaks to gene promoters were determined using ChIPseeker (74).

### Analysis of transcription read-through and differential gene expression.

The numbers of read fragments per gene were determined from the mapped 4sU-seq and RNA-seq reads in a strand-specific manner using featureCounts (75) and gene annotations from Ensembl (v87 for GRCh37/hg19) (76). All fragments (read pairs for paired-end sequencing or reads for single-end sequencing) overlapping exonic regions on the corresponding strand by ≥25 bp were counted for the corresponding gene. Expression of protein-coding genes and lincRNAs was quantified in terms of fragments per kilobase of exon model per million mapped fragments (FPKM) and averaged between replicates. Only fragments mapping to the human genome were counted for the number of mapped fragments, as described previously (20). Downstream and upstream transcription for genes was determined from 4sU-seq data, as described previously (21), i.e., the FPKM in the 5-kb windows down- or upstream of genes divided by the gene FPKM. Readthrough transcription was quantified as the difference in downstream transcription between infected and uninfected cells, with negative values set to zero. Read-in transcription was calculated analogously as the difference in upstream transcription between infected and uninfected cells. For full details, see our previous publication (21). Only genes were included here that (i) had no upstream or downstream gene within 5 kb, (ii) were expressed (FPKM ≥ 1 in 4sU-RNA) in uninfected cells or at least one time point of WT infection, and (iii) had at most 10% read-in transcription at any time during WT infection. For genes not expressed in uninfected cells (FPKM < 1 in uninfected 4sU-RNA), at most 5% read-in transcription during infection and at most 25% upstream transcription in uninfected cells was allowed. These restrictions were used to exclude genes that only appeared induced due to read-in transcription from an upstream gene. In total, 4,162 genes were included for the analyses described here. Differential gene expression analysis for these genes in total RNA, 4sU-RNA, and subcellular RNA fractions was performed based on gene read counts using DESeq2 (24), and *P* values were adjusted for multiple testing using the method by Benjamini and Hochberg (68). Additional candidate upregulated genes with low or no expression in uninfected cells were determined using the following criteria: (i) FPKM in uninfected 4sU-RNA and total RNA  ≤ 1; (ii) FPKM in either 4sU-RNA or total RNA at any time of infection both ≥0.5- and ≥4-fold higher than in uninfected cells; and (iii) a read-in transcription of ≤20% at all time points. Candidate genes were subsequently validated by manual inspection of mapped reads for individual replicates in the IGV genome browser (77). To identify the extended set of genes transcriptionally downregulated in a *vhs*-dependent manner, we used DESeq2 to calculate fold changes for all genes between chromatin-associated RNA at 8 h after WT infection versus mock infection and 8 h after *Δvhs* infection versus mock infection. Genes were defined as transcriptionally downregulated in a *vhs*-dependent manner if they were significantly downregulated in WT (log_2_-fold change ≤ –1, adj. *P* ≤ 0.001), not downregulated in Δ*vhs* infection (log_2_-fold change > −1), and there was at least a 2-fold increase in fold changes in Δ*vhs* infection compared to WT infection.

### RNA half-lives.

RNA half-lives were measured as described previously (28) from 4sU-RNA and total RNA measurements in uninfected cells from the WT time course. For this purpose, we first calculated the ratio of gene expression (FPKM) values in 4sU-RNA versus total RNA in uninfected cells for all genes, normalized this ratio assuming a median RNA half-life of 5 h to determine the fraction of RNA newly transcribed in 1 h for each gene [N*(*t*), *t *= 60], and then calculated the RNA half-life for each gene as *t*_1/2_ = –*t*ln2/ln(1 – N*(*t*)).

### Mathematical model.

The ODE model of WT and Δ*vhs* infections is described in Text S2 in the supplemental material.

### Clustering, enrichment, and network analysis.

Hierarchical clustering was performed in R (78) using Euclidean distances and Ward’s clustering criterion (79). Gene Ontology (GO) (29) annotations for genes were obtained from EnrichR (80), and lists of IFN I, II, and III up- or downregulated genes (at least 2-fold) were obtained from the INTERFEROME database (30). Genes regulated by doxycycline-inducible DUX4 were taken from the study of Jagannathan et al. (upregulated, log_2_-fold change  ≥ 1, FDR  ≤ 0.001; downregulated, log_2_-fold change ≤ −1, FDR  ≤ 0.001) (51). TCF-dependent genes and SRF targets in MEFs were taken from the study by Gualdrini et al. (42). Odds ratios and the significance of enrichment compared to the background of 4,162 genes were determined by using the Fisher exact test in R (78), and *P* values were adjusted for multiple testing using the method by Benjamini and Hochberg (68). Human protein-protein associations were downloaded from the STRING database (39) (v10.5) using NDEx (81) and visualized in Cytoscape (82). Only associations with a score of ≥350 are shown.

### Comparison of muSOX and *vhs*-dependent genes.

Fold changes for WT and ΔHS MHV68 infection were taken from the study of Abernathy et al. (18) and downloaded from Gene Expression Omnibus (GEO; GSE70481). Mouse and human gene symbols were mapped to their orthologues in the respective other species using the Mouse/Human orthology table from the Mouse Genome Informatics (MGI) database (83). muSOX-dependent genes were defined according to the criteria applied by Abernathy et al.: downregulated in WT (log_2_-fold change ≤ −1 and FDR  ≤ 0.1) but not in ΔHS infection (log_2_-fold change > −1 or FDR  > 0.1). *vhs*-dependent genes were defined according to our criteria described above.

### Transcription factor binding motif search.

Promoter motif search for *vhs*-dependently downregulated genes was performed using HOMER in proximal promoter regions (bp −2,000 to +2,000 relative to the transcription start site) (84). Potential transcription binding factor sites in uninfected cells were further identified by using ATAC-seq (assay for transposase-accessible chromatin using sequencing [85]) data for uninfected cells from our previous study (*n* = 2 replicates) (21). ATAC-seq data were mapped against hg19, as previously described (21), and open chromatin peaks were determined using MACS2 (86). Blacklisted regions for hg19 (accession number ENCFF001TDO) were downloaded from ENCODE (87), and peaks called in regions overlapping with blacklisted regions were removed from further analysis. Furthermore, only peaks occurring in both replicates were considered for motif search. A motif search was then performed using HOMER for open chromatin peaks within 10, 25, and 50 kb, respectively, of *vhs*-dependently downregulated genes.

### Data availability.

All sequencing data are available in the GEO database under the following IDs: 4sU-seq and total RNA-seq data of WT infection, GSE59717; 4sU-seq and total RNA-seq data of Δ*vhs* infection, GSE129715; RNA-seq of total, cytoplasmic, nucleoplasmic, and chromatin-associated RNA in WT and Δ*vhs* infections, GSE129582; RNA-seq of chromatin-associated RNA in WT, Δ*vhs*, vhs D195N, and WT-BAC infections, GSE140068; and H3K4me3 ChIPmentation, GSE132920.

## Supplementary Material

Supplemental file 1

Supplemental file 2

Supplemental file 3

Supplemental file 4

Supplemental file 5

Supplemental file 6

Supplemental file 7

Supplemental file 8

Supplemental file 9
