# Supplementary material for: Dissecting Herpes Simplex Virus 1-Induced Host Shutoff at the RNA Level
Source: J Virol. 2021 Jan 13;95(3):e01399-20. doi: 10.1128/JVI.01399-20 (PMC7925104; doi:10.1128/JVI.01399-20)
Supplement: Supplemental file 2 [file JVI.01399-20-s0002.pdf]

S2 Text: Mathematical model of *vhs* activity and loss of transcriptional activity in HSV-1 infection

Model network

In our model (see network diagram below), we distinguish between nuclear RNA $N(t)$ and cytoplasmic RNA $C(t)$. Nuclear RNA is newly transcribed with rate $s(t)$, degraded with rate $k = \frac{\ln 2}{t_{1/2}}$ ($t_{1/2}$ = RNA half-life), and exported with rate ε. The degradation rate k does not change over time and reflects the "normal" RNA turnover in the cell. We assume that ε also stays constant over time and is the same for all genes considered. Furthermore, we assume that *vhs* activity is limited to the cytoplasm and that nuclear RNA is protected from *vhs*-mediated decay. In contrast, cytoplasmic RNA is both degraded at the normal rate k as well as by *vhs*-mediated decay at the rate $v(t)$.

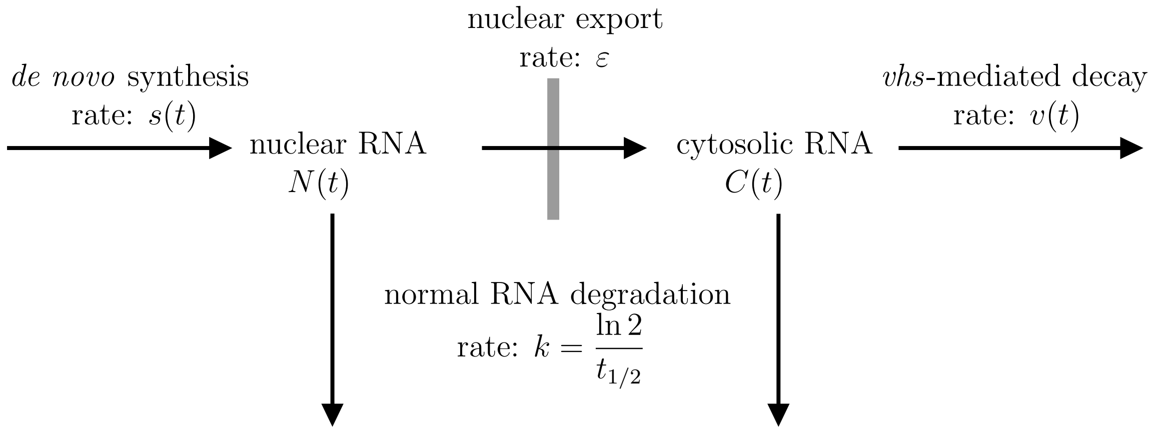

ODE model

The following ordinary differential equations (ODEs) describe the change over time for nuclear RNA $N(t)$, cytosolic RNA $C(t)$ and total RNA $T(t) = N(t) + C(t)$:

Change in nuclear RNA $N(t)$:

$$\frac{dN}{dt} = \underbrace{s(t)}_{\text{synthesis}} - \underbrace{kN}_{\substack{\text{normal}\\\text{degradation}}} - \underbrace{\varepsilon N}_{\text{export}} \tag{1}$$

Change in cytosolic RNA $C(t)$:

$$\frac{dC}{dt} = \underbrace{\varepsilon N}_{\text{export}} - \underbrace{kC}_{\substack{\text{normal}\\\text{degradation}}} - \underbrace{v(t)C}_{\substack{\textit{vhs}\text{-mediated}\\\text{degradation}}} \tag{2}$$

Change in total RNA $T = N + C$:

$$\begin{aligned}\frac{dT}{dt} &= \frac{dN}{dt} + \frac{dC}{dt}\\ &= s(t) - kN - \varepsilon N + \varepsilon N - kC - v(t)C\\ &= s(t) - k(N + C) - v(t)C\\ &= s(t) - kT - v(t)C\end{aligned} \tag{3}$$

Results of the model for uninfected cells

In uninfected cells, i.e. steady state, there is no change in total RNA concentrations, i.e. $\frac{dT}{dt} = 0$ and $T(t) = T(0) =: T_0$. Furthermore, $v(t) = 0$. This allows calculating the synthesis rate in uninfected cells as a function of the degradation rate k:

$$\begin{aligned}\frac{dT}{dt} &= s(t) - kT = 0\\ \Rightarrow s(t) &= kT(t) = kT_0\end{aligned} \tag{4}$$

Furthermore, both nuclear and cytosolic RNA levels also remain constant, i.e. $N(t) = N(0) =: N_0$ and $C(t) = C(0) =: C_0$.

$$\begin{aligned}&\Rightarrow \frac{dN}{dt} = kT_0 - kN - \varepsilon N = 0\\ &\Rightarrow N_0 = N(t) = \frac{k}{k+\varepsilon}T_0\\ &\Rightarrow C_0 = C(T) = \frac{\varepsilon}{k+\varepsilon}T_0\end{aligned} \tag{5}$$

Thus, for each gene the fractions of nucleic and cytoplasmic RNA depend on the normal degradation rate k for this gene and the global export rate ε.

Results of the model for Δ*vhs* infection

In Δvhs infection, there is no *vhs*-mediated decay, i.e. $v(t) = 0$. Since this is the easier scenario than wild-type (WT) infection, we will first focus on this case. We then have

$$\frac{dT}{dt} = s(t) - kT \tag{6}$$

In the following, we assume that the synthesis rate $s(t)$ in infected cells is proportional to the synthesis rate in uninfected cells, i.e. $s(t) \propto kT_0$ (see equation 4). Furthermore, $s(t)$ depends both on a gene-specific fold-change in transcription $\delta(t)$ ($\delta(t) > 0$), which varies between genes, and a genome-wide drop in transcriptional activity $s^*(t)$ ($0 < s^*(t) < 1$), which is the same for all genes. $\delta(t)$ allows modeling transcriptional up- ($\delta(t) > 1$) or down-regulation ($\delta(t) < 1$) of individual genes. Furthermore, the smaller $s^*(t)$, the higher is the global drop in transcriptional activity.

Thus, $s(t)$ is defined in the following way for both Δvhs and WT infection:

$$s(t) = \delta(t)s^*(t)kT_0. \tag{7}$$

To model the genome-wide drop in transcriptional activity, we define $s^*(t)$ as

$$s^*(t) := \frac{\rho + e^{-\sigma t}}{1 + \rho} \tag{8}$$

Here, σ should be > 0, because otherwise $s^*(t)$ would increase over time. We then have that

$$\lim_{t \to \infty} s^*(t) = \frac{\rho}{1 + \rho}. \tag{9}$$

This means that for large t, $s^*(t)$ approaches $\frac{\rho}{1+\rho}$. Thus, ρ should be chosen such that $0 < \frac{\rho}{1+\rho} < 1$, i.e. $\rho > 0$.

This definition allows modeling a range of different behaviors, including an exponential drop quickly reaching an almost constant value (for very large σ) as well as an almost linear drop (for very small σ) for the time interval considered (see Fig. 1 A,C,E,G).

To obtain a specific drop s_e in transcriptional activity by the end of the time-course t_e, i.e. $s^*(t_e) = s_e$, set

$$\rho = \frac{s_e - e^{-\sigma t_e}}{1 - s_e}. \tag{10}$$

If $\delta(t) = 1$ (i.e. no gene-specific regulation), the solution for $T(t)$ is

$$T(t) = \frac{\rho T_0}{1+\rho} + \frac{kT_0}{(1+\rho)(k-\sigma)}e^{-\sigma t} - \frac{\sigma T_0}{(1+\rho)(k-\sigma)}e^{-kt} \quad (11)$$

Thus, the fold-change in total mRNA levels is

$$\frac{T(t)}{T_0} = \frac{\rho}{1+\rho} + \frac{k}{(1+\rho)(k-\sigma)}e^{-\sigma t} - \frac{\sigma}{(1+\rho)(k-\sigma)}e^{-kt} \quad (12)$$

Figure 1 illustrates total RNA log2 fold-changes obtained at 8h p.i. using this model for genes with different mRNA half-lives, i.e. different degradation rates. For this purpose, we used the mRNA half-lives determined from total and newly transcribed (4sU-) RNA in uninfected cells for the 4,162 genes included in our analysis (see main manuscript). Different values of s_e and σ were evaluated for $s^*(t)$. Subfigures on the left-hand side show $s^*(t)$. Subfigures on the right-hand side show RNA half-lives plotted against the total RNA log2 fold-changes, i.e. $\log_2\left(\frac{T(t)}{T_0}\right)$, obtained for this choice of $s^*(t)$.

Variance in total RNA log2 fold-changes for each choice of $s^*(t)$ are indicated in the legend for comparison against the variance observed in the experimental data (see also Table 1). As variance is scale-invariant and thus independent of whether absolute or relative fold-changes are considered, it can be compared between our model, which determines absolute fold-changes, and the analysis of the experimental data, which is focused on relative fold-changes.

The following observations can be made from these results:

1. Total RNA fold-changes decrease continuously with decreasing mRNA half-life, i.e. unstable mRNAs are more strongly down-regulated. This is observed for any choice of s_e and σ.

2. At the same s_e, higher values of σ, i.e. faster drops in transcriptional activity, lead to lower total RNA fold-changes (i.e. stronger down-regulation) as well as a higher variance in total RNA log2 fold-changes.

3. With decreasing s_e, lower total RNA fold-changes (i.e. stronger down-regulation) as well as a higher variance in total RNA log2 fold-changes is observed.

Thus, both the speed of the decrease in transcriptional activity as well as how strongly it is reduced at the end of the time-course influence the extent of the observed down-regulation and the variance in log2 fold-changes. However, the rank correlation r_s between mRNA half-life and log2 fold-changes is independent of the

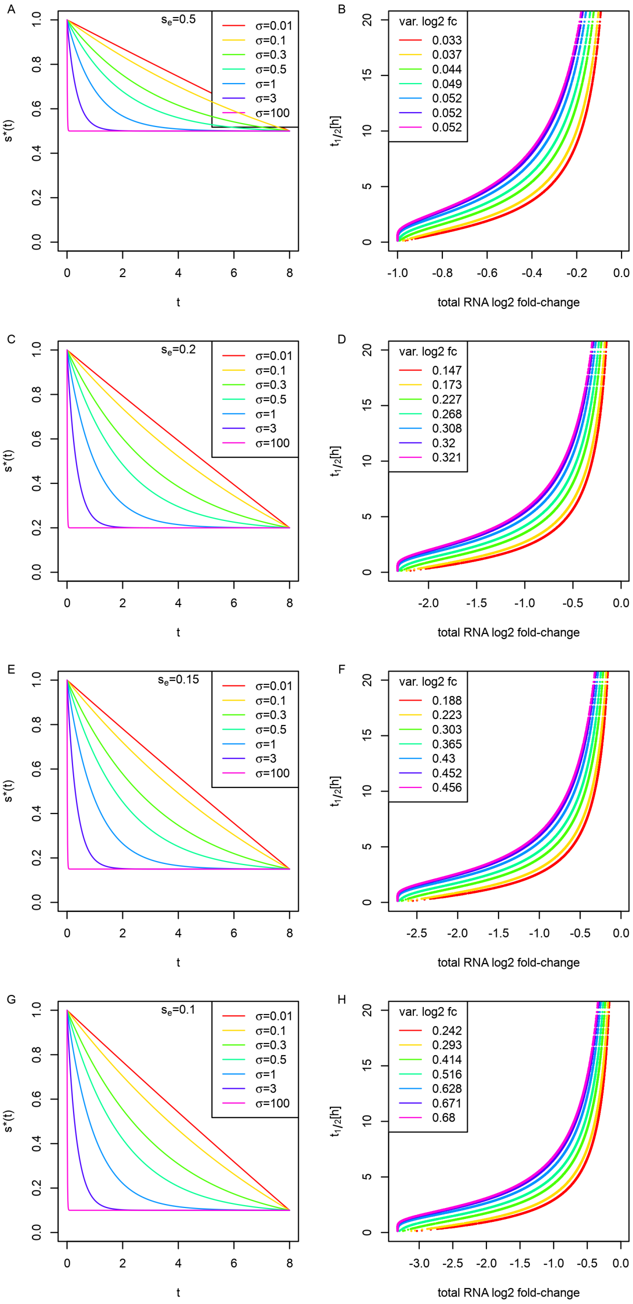

Fig 1: Results of the model for Δvhs infection for different values for different choices of $s^*(t)$. On the left-hand side, $s^*(t)$ is shown for different values of s_e and σ. On the right-hand side, total RNA log2 fold-changes at 8h p.i. (x-axis) obtained with our model for this choice of $s^*(t)$ are plotted against RNA half-lives $t_{1/2}$ (y-axis). Each row of subfigures corresponds to one value of s_e (A,B: $s_e = 0.5$, C,D: $s_e = 0.2$, E,F: $s_e = 0.15$, G,H: $s_e = 0.1$). Each line in the subfigures corresponds to one value of σ (color code indicated in subfigures on the left). Variance of total RNA log2 fold-changes for a specific choice of $s^*(t)$ is indicated in the legend of the right-hand side subfigures.

parameter choice, i.e. we always have $r_s = 1$. The lower correlation observed in the real-life experiments is likely due to both noise as well as gene-specific changes in transcription for individual genes due to transcriptional up- or down-regulation.

To estimate both the extent of the drop in transcriptional activity as well as the speed of the drop, we compared the variance in total RNA log2 fold-changes at 2, 4, 6 and 8h p.i. between the RNA-seq Δvhs infection time-course (Table 1) and the results of our model for different parameter values. The high variance observed at 8h p.i. in the RNA-seq data could only be obtained in our model with a dramatic drop in transcriptional activity to 20% of the original level or lower ($s_e \leq 0.2$) at 8h p.i. Furthermore, the relatively low variance at the beginning of the time-course suggests a more gradual drop in transcriptional activity (the yellow and light green curves in Fig. 1) rather than a very quick drop (violet and pink curves). With such a more gradual decrease, however, the large variance in fold-changes observed at 8h p.i. can only be obtained in our model with an even stronger drop in transcriptional activity ($s_e \leq 0.1$). In summary, our modeling results suggest a very pronounced drop in transcriptional activity to as little as 10% of the original level at 8h p.i.

**time point Δvhs infection**	**variance log2 fold-change (total RNA)**
2h p.i.	0.002
4h p.i.	0.05
6h p.i	0.19
8h p.i.	0.451

Table 1: Variance in total RNA log2 fold-changes for the total RNA-seq time-course of Δvhs infection described in the main manuscript.

Since a larger variance in total RNA log2 fold-changes could also be obtained by gene-specific transcriptional regulation, we also analyzed the effect of including gene-specific regulation using the function $\delta(t)$. Here, we used fold-changes obtained from chromatin-associated RNA at 8h p.i. Δvhs infection (denoted as $fc_{chr}(t_e)$) to model the extent of transcriptional regulation. Since fold-changes in chromatin-associated RNA were not available for earlier time-points, we modeled the change in regulation over time by assuming a linear increase or decrease in log2 fold-changes from 0 to 8h p.i. Thus, for each gene

$$\log_2(\delta(t)) = \frac{t}{t_e} \log_2(fc_{chr}(t_e)). \quad (13)$$

This means that $\delta(t) = fc_{chr}(t_e)^{\frac{t}{t_e}}$.

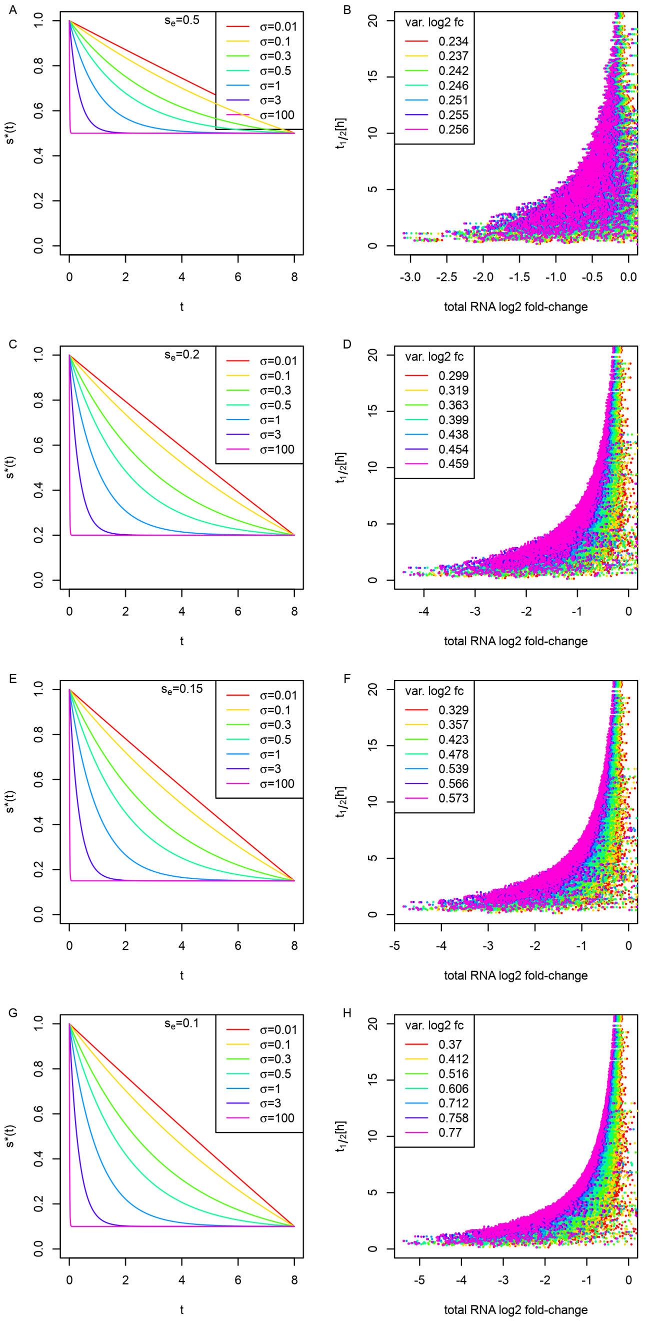

Fig 2: Results of the model for Δvhs infection with gene-specific transcriptional up- or down-regulation and different values of s_e and σ. $\delta(t)$ was determined as indicated in equation 13. See Fig. 1 for description of subfigures.

Results of the model for this choice of $\delta(t)$ are shown in Fig. 2. Notably, we now obtained correlation coefficients $r_s < 1$ between mRNA half-life and modeled total RNA log2 fold-changes. Furthermore, r_s was smaller for slower rates of decrease, i.e. lower σ, than for faster drops. Although transcriptional regulation tended to increase the variance in total RNA log2 fold-changes for higher values of s_e, observed variances were still more consistent with a large overall drop in transcriptional activity ($s_e \leq 0.2$) with a more gradual rate of decrease ($\sigma \leq 0.3$). In summary, our mathematical model provides evidence that overall transcriptional activity in Δ*vhs* infection is reduced to ≤20% of the transcriptional activity in uninfected cells (see also Fig. 6 A).

Results of the model for WT infection

Next, we evaluated the model for WT HSV-1 infection, which combines the drop in transcriptional activity with *vhs*-mediated decay. For this purpose, we used the same definition of $s(t)$ as in equation 7 and the following definition of $v(t)$:

$$v(t) = \lambda(1 - e^{-\mu t}), \tag{14}$$

with $\mu, \lambda > 0$.

Again, this allows modeling both the final decay rate at the end of the time-course as well as the speed in which this decay rate is reached (see Fig. 3 A for an example). To obtain a decay rate $v(t_e) = v_e$ at the end of our time-course t_e for a given μ, λ should be chosen such that

$$\begin{aligned} v_e = v(t_e) =& \lambda(1 - e^{-\mu t_e}) \\ \iff \lambda =& \frac{v_e}{1 - e^{-\mu t_e}} \end{aligned} \tag{15}$$

From a given value for $v(t)$, we can calculate the fraction of RNA lost per hour due to *vhs*-mediated as

$$1 - e^{-v(t)}. \tag{16}$$

We obtained numerical solutions for our model for different parameter combinations (see Table 2) to evaluate the effect on the correlation between total RNA log2 fold-changes and RNA half-life. For instance, in the example shown in Fig. 3 B, negative correlations are observed for any choice of μ. This is due to the fact that in this example we used (i) a high final decay rate ($v_e = 0.7$), such that 50% of RNAs are lost due to *vhs* activity after as little as 1h, and (ii) a relatively high export rate such that around 85% of RNA is cytosolic for a gene with median RNA half-life of 5h. The latter means that a large fraction of RNA is susceptible to *vhs*-mediated decay.

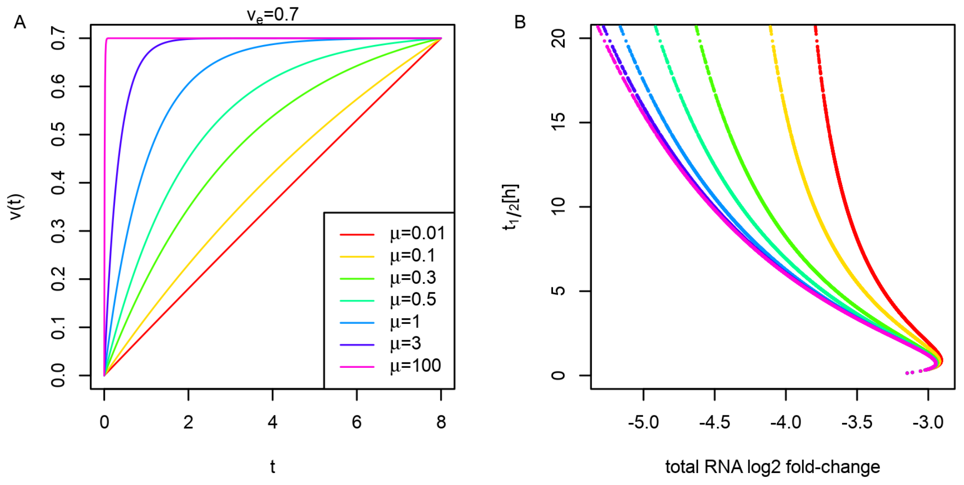

Fig 3: Results of the model with *vhs*-mediated decay for example parameter combinations. (A) A maximum decay rate of $v_e = 0.7$ is modeled with different values of μ. For small μ, we observe an almost linear increase, while for large μ v_e is reached almost immediately. (B) Results of the model for parameter values in (A), $\sigma = 0.1$ and $s_e = 0.1$ (i.e. a gradual drop with highly reduced transcriptional activity (only 10% of original) at the end of the time-course) and export rate $\varepsilon = 0.8$. At this export rate, a gene with an RNA half-life of 5h (i.e. median RNA half-life) will have approximately 85% cytosolic RNA in steady state, i.e. uninfected cells.

Resulting correlation coefficients at 8h p.i. without gene-specific regulation are illustrated for all parameter combinations in Fig. 4. Here, almost all combinations resulted either in correlation coefficients of 1 (for low values of v_e, i.e. 0.175 and 0.088) or -1 (for high values of v_e, i.e. 0.70 and 0.35), mostly independent of the choice of μ. Only few showed correlation coefficients in the range observed in the experimental data (indicated by gray background).

Interestingly, however, this picture changed dramatically when taking into account gene-specific transcriptional regulation obtained from log2 fold-changes in chromatin-associated RNA at 8h p.i. as described in equation 13 (Fig. 5). In this case, a wider range of correlation coefficients was observed depending on the value of μ. Nevertheless, negative correlation coefficients were only observed for high values of v_e (i.e. $v_e = 0.7$ or $v_e = 0.35$).

A range of parameter combinations resulted in correlation coefficients in the approximate range observed in our experimental data ($r_s = -0.31$ at 2h p.i., $r_s = -0.37$ at 4h and 6h p.i. and $r_s = -0.38$ at 8h p.i.). However, only one of the evaluated parameter combinations resulted in correlation coefficients that were within 5% of the observed correlation coefficients at all 4 time points, i.e. $v_e = 0.35, \mu = 1, \varepsilon = 0.8, s_e = 0.2$ and $\sigma = 0.3$. Fig. 6 A illustrates the drop in

param.	evaluated values	motivation for choice
s_e	$0.1, 0.2$	Upper bounds obtained from the analysis on Δvhs infection
σ	$0.1, 0.3$	Likely values obtained from the analysis on Δvhs infection
v_e	$0.70, 0.35, 0.175, 0.088$	Decay rate at $t_e = 8$h chosen such that 50%, 30%, 16%, 8% of RNA, respectively, are lost within 1h due to *vhs*-mediated decay (see equation 16).
μ	$0.01, 0.1, 0.3, 0.5, 1, 3, 100$	Models different rates of increase in *vhs*-mediated decay, from an almost linear increase ($\mu = 0.01$) to almost constant decay rates ($\mu = 100$, see Fig. 3 A)
ε	$0.2, 0.3, 0.8, 3$	In steady-state, this results in approximately 59, 68, 85 and 96% of RNA, respectively, being cytoplasmic (see equation 5) for a gene with RNA half-life of 5h, i.e. median RNA half-life in our analysis.

Table 2: Parameters evaluated for the model of WT infection.

transcriptional activity obtained with these values of s_e and σ. This corresponds to a gradual but faster than linear drop down to 20% of the original transcriptional activity. Fig. 6 B illustrates the percentage of mRNAs lost per hour by *vhs*-mediated decay for $v_e = 0.35$ and $\mu = 1$. This suggests a quick increase of *vhs* activity with approximately 26% of RNAs lost per hour due to *vhs* activity already at 2h p.i., followed by a stabilization with around 30% of RNAs lost per hour from 4h p.i. to 8h p.i.

Since the definition of $v(t)$ used for this analysis does not allow for a decrease in *vhs* activity, we also evaluated an alternative model in which *vhs* activity increased only until time point $t_{\max}$ and then decreased again. For this purpose, *vhs* activity is modeled by the function $v^*(t)$ with

$$v^*(t) = \begin{cases} v(t), & \text{if } t \leq t_{\max} \\ u(t), & t > t_{\max} \end{cases} \tag{17}$$

and

$$u(t) = \psi\left(1 - e^{\phi(t-t_e)}\right). \tag{18}$$

Here, $\phi > 0$ and $t_e = 8$h the end of the time-course. Furthermore $\psi > 0$ is defined

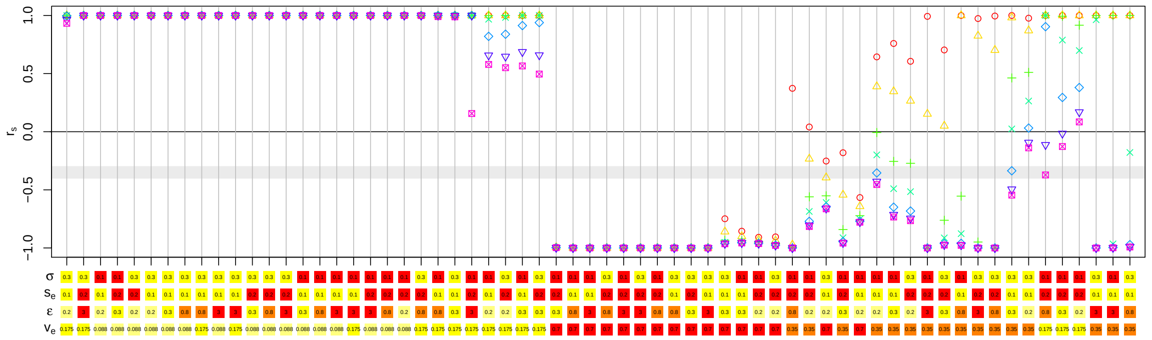

Fig 4: Summary of correlation coefficients between total RNA log2 fold-changes and RNA half-lives for the model of WT HSV-1 infection without gene-specific transcriptional regulation. Colors of symbols indicate the value of μ (see Fig. 3 A and Table 2) and colored squares below the x-axis show values for the parameters σ, s_e, ε and v_e (red = highest values, yellow = lowest values). Combinations of σ, s_e, ε and v_e are clustered based on the similarity of correlation coefficients for the different values of μ (using Euclidean distances and hierarchical clustering with Ward's minimum variance method). Gray background highlights correlation coefficients between -0.4 and -0.3, which is broadly the range of correlation coefficients observed in the experimental data for WT HSV-1 infection.

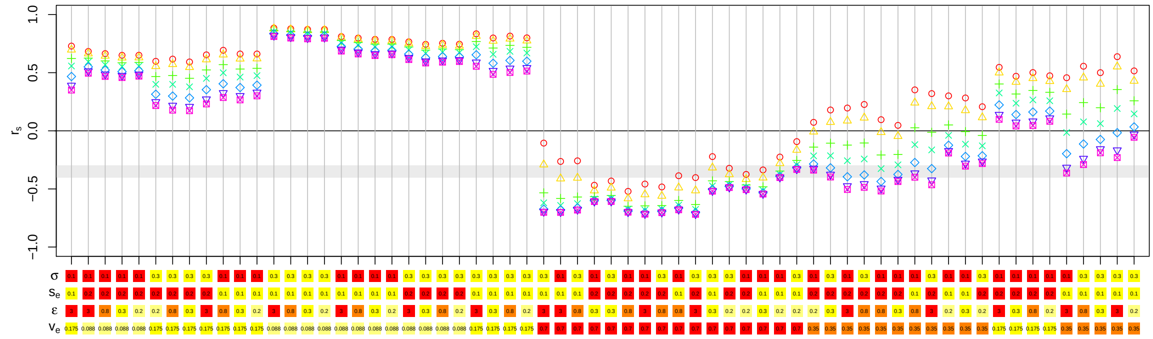

Fig 5: Summary of correlation coefficients between total RNA log2 fold-changes and RNA half-lives obtained with the model of WT HSV-1 infection with gene-specific transcriptional regulation. For detailed description see Fig. 4.

such that $v(t_{\max}) = u(t_{\max})$. This means that

$$\psi = \lambda(1 - e^{-\mu t_{\max}})/(1 - e^{\phi(t_{\max} - t_e)}). \quad (19)$$

By choosing different values of ϕ, different rates of decrease after $t_{\max}$ can be modeled. Small values of ϕ lead to an almost linear decrease in $v^*(t)$ after $t_{\max}$, while large values of ϕ result in essentially constant values for $v^*(t)$ until t_e (see Fig. 7 A).

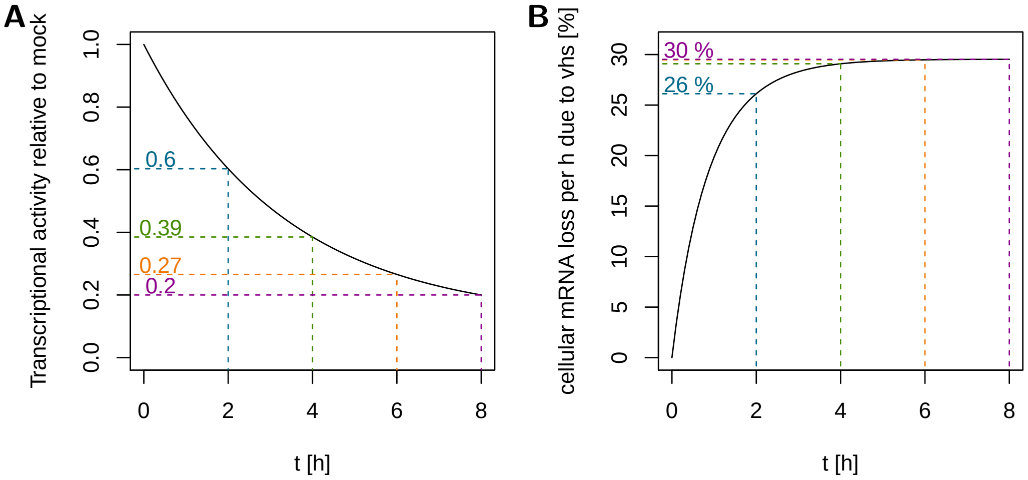

Fig 6: Predictions of the model for the parameter combinations that resulted in correlation coefficients between total RNA log2 fold-changes and RNA half-life within 5% of the observed values at 2, 4, 6 and 8h p.i. ($v_e = 0.35, \mu = 1, \varepsilon = 0.8, s_e = 0.2$ and $\sigma = 0.3$). (A) Transcriptional activity relative to uninfected cells (mock) for the first 8h of infection. (B) Percentage of RNA lost due to *vhs*-mediated decay per hour for the first 8h of infection. This figure is also shown in the main manuscript.

To evaluate whether decreasing *vhs* activity later in infection is consistent with the correlation coefficients observed in the experimental data, we evaluated $t_{\max} \in \{2, 4, 6\}$ (i.e. a decrease in *vhs* activity starting at 2, 4 or 6h p.i.) and the same values for ϕ as previously evaluated for μ (see Table 2 and Fig. 7). For the remaining parameters of the model (σ, s_e, ε, v_e, μ), we only evaluated parameter combinations that resulted in correlation coefficients for 2h p.i. within 5% of the observed value (see Table 3).

Results are illustrated in Fig. 7 B. This shows that a decrease in *vhs* activity after time $t_{\max}$ always lead to an increase in correlation coefficients r_s between total RNA fold-changes and RNA half-lives, i.e. r_s became less negative or positive at all time points after $t_{\max}$. This is a consequence of the increasing reduction in transcriptional activity, which becomes more dominant than *vhs*-mediated decay if *vhs* activity decreases.

The only parameter combinations that resulted in correlation coefficients within 5% of the observed values for all time points were $v_e = 0.35, \mu = 1, s_e = 0.2, \sigma = 0.3$ and $\varepsilon = 0.8$, i.e. the same parameters that yielded correlation coefficients within the 5% range for the model without the decrease in *vhs* activity. Furthermore, $t_{\max}$ was 4 or 6 and $\phi = 100$. This corresponds to the pink curve in Fig. 7 A and means that from either 4 or 6 hours *vhs* activity remains approximately

selected parameter combinations					match for	
v_e	μ	s_e	σ	ε	$t_{\max}$	ϕ
0.175	100	0.2	0.1	3	–	–
0.175	3	0.1	0.1	0.8	–	–
0.35	1	0.2	0.1	3	–	–
0.35	1	0.2	0.3	0.8	4 or 6	100
0.7	0.1	0.1	0.1	0.3	–	–

Table 3: The table shows parameter combinations for σ, s_e, ε, v_e and μ evaluated in combination with a reduction in *vhs* activity after $t_{\max} = 2, 4$ or 6h p.i. Parameter combinations were chosen from the values in Table 2 such that correlation coefficients at 2h p.i. were within 5% of the observed correlation coefficients. The last two columns show parameter values for $t_{\max}$ and ϕ that led to correlation coefficients for all time points within 5% of the observed correlation coefficients. "–" indicates that none of the evaluated values for $t_{\max}$ and ϕ resulted in matching correlation coefficients for all time points.

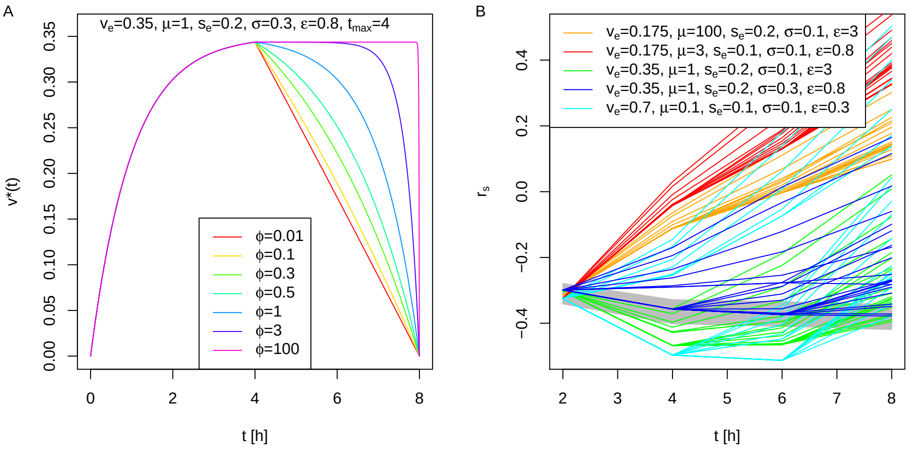

Fig 7: (A) Example curves for $v^*(t)$ modeling the decrease of *vhs* activity after $t_{\max} = 4$h using different values for ϕ. Other parameter values are indicated on the top of the figure. (B) Correlation coefficients r_s between total RNA log2 fold-changes and RNA half-lives obtained at 2, 4, 6 and 8h p.i. for different parameter choices for $v^*(t)$. Each line represents one choice of $t_{\max}$ and ϕ. Lines are color-coded according to the value of σ, s_e, ε, v_e and μ (see legend). Grey background indicates the range of r_s within 10% of the r_s values observed in the real data.

constant until 8h p.i. In the model without the decrease in *vhs* activity, *vhs* activity also barely changes between 4 and 8 hours (see Fig. 6 B). Thus, all parameter

combinations resulting in correlation coefficients within 5% of observed values for all time points correspond to a rapid increase in *vhs* activity until approximately 4h, followed by stable activity until 8h p.i. Even if we allow a 10% deviation, the only other parameter combination resulting in close enough correlation coefficients were $t_{\max} = 6$ and $\phi = 3$ (all other parameters the same). This corresponds to a very late reduction in *vhs* activity after 7h p.i. In summary, the results of the model indicate that *vhs* activity increases quickly within the first 2h p.i., further increases moderately until 4h p.i. and then remains approximately constant.

S10 Text: H3K4me3 ChIPmentation

Two days prior to harvesting, 1.75e6 HFF cells were seeded in 15cm dishes. At the day of harvest, cells had expanded to ~80% confluency. Mock and HSV-1 infected samples (8h p.i.) were fixed by adding formaldehyde to a final concentration of 1% and incubating for 10 minutes at 37℃. Fixation was stopped by adding glycine to a final concentration of 125mM for 5 minutes at room temperature. After fixation, medium was aspirated and cells were washed twice with ice-cold PBS. Cells were scraped in 1mL of ice-cold PBS containing protease inhibitor cocktail (1x) (Roche #11836153001) with an additional 1mM phenylmethylsulfonyl fluoride (PMSF). Cells were collected in 1.5 mL tubes (each plate in a separate tube) and pelleted at 1500 rpm, 20 min, 4℃. Supernatant was aspirated and cell pellets were frozen in liquid N_2.

Cell pellets were resuspended in 1.5 mL 0.25% SDS sonication buffer (10mM Tris pH=8.0, 0.25% [w/v] SDS, 2mM EDTA) with 1x protease inhibitors and 1mM additional PMSF and incubated on ice for 10 minutes. Cells were sonicated in seven 1 minute intervals, 25% amplitude, with *Branson Ultrasonics Sonifier™ S-450* until most fragments were in the range of 200-700 bp. 500,000 cells used for the preparation of the ChIPmentation libraries were diluted 1:1.5 with equilibration buffer (10 mM Tris, 233mM NaCl, 1.66% [v/v] Triton X-100, 0.166% [w/v] sodium deoxycholate, 1mM EDTA, protease inhibitors) and spun at 14,000x g, 4℃ for 10 minutes to pellet insoluble material. Supernatant was transferred to a new 1.5 mL screw-cap tube and topped up with RIPA-LS (10mM Tris-HCl pH 8.0, 140mM NaCl, 1mM EDTA pH 8.0, 0.1% [w/v] SDS, 0.1% [w/v] sodium deoxycholate, 1% [v/v] Triton X-100, protease inhibitors) to 600 µL. Input and gel samples were preserved and later on subjected to de-crosslinking and DNA isolation.

Lysates were incubated with anti-H3K4me3 antibody (Diagenode #C15410003-50) on a rotator overnight at 4℃. Dependent on the added amount of antibody, the amount of Protein G magnetic beads (ThermoFisher) was adjusted (e.g. for 1-2 µg of antibody/IP = 15 µL of beads) and blocked overnight with 0.1% [w/v] bovine serum albumin in RIPA buffer. On the following day, beads were added to the IP samples for 2 hours on a rotator at 4℃ to capture the antibody-bound fragments. The immunoprecipitated chromatin was subsequently washed twice with 150 µL each of ice-cold buffers RIPA-LS, RIPA-HS (10mM Tris-HCl pH 8.0, 500mM NaCl, 1mM EDTA pH 8.0, 0.1% [w/v] SDS, 0.1% [v/v] sodium deoxycholate, 1% [v/v] Triton X-100), RIPA-LiCl (10mM Tris-HCl pH 8.0, 250mM LiCl, 1mM EDTA pH 8.0, 0.5% [w/v] sodium deoxycholate, 0.5% [v/v] Nonidet P-40) and 10mM Tris pH 8.0 containing protease inhibitors. Beads were washed once more with ice-cold 10mM Tris pH 8.0 lacking inhibitors and transferred into new tubes.

Beads were resuspended in 25 µL of the tagmentation reaction mix (Nextera DNA Sample Prep Kit (Illumina)) containing 5 µL of 5x Tagmentation buffer, 1 µL of Tagment DNA enzyme, topped up with H_2O to the final volume and incubated at 37℃ for 10 minutes in a thermocycler. Beads were mixed after 5 minutes by gentle pipetting. To inactivate the Tn5 enzyme, 150 µL of ice cold RIPA-LS was added to the tagmentation reaction. Beads were washed twice with 150 µL of RIPA-LS and 1x Tris-EDTA and subjected to de-crosslinking by adding 100 uL ChIPmentation elution buffer (160mM NaCl, 40 µg/mL RNase A, 1x Tris-EDTA [Sigma #T9285| and incubating for 1h at 37℃ followed by overnight shaking at 65℃. The next day, 4mM EDTA and 200 µg/mL Proteinase K were added and samples incubated for another 2h at 45℃ with

1000 rpm shaking. Supernatant was transferred into a new tube and another 100 µL of ChIPmentation elution buffer was added for another hour at 45℃ with 1000 rpm shaking. DNA was isolated with MinElute PCR Purification Kit (Qiagen, #28004) and eluted in 21 µL of H_20.

DNA for the final library was prepared with 25 µL NEBNext Ultra II Q5 Master Mix, 3.75 µL IDT custom primer i5_n (10 µM, see below); 3.75 µl IDT custom primer i7_n (10 µM, see below); 3.75 µL PPC (PCR Primer Cocktail, Illumina) (10 µM), 13.75 µL ChIPmentation DNA. The Cq value obtained from the library quantification, rounded up to the nearest integer plus one additional cycle, was used to amplify the rest of the ChIPmentation DNA. Library qualities were verified by High Sensitivity DNA Analysis on the Bioanalyzer 2100 (Agilent) before performing sequencing on NextSeq 500 (paired-end 35bp reads) at the University of Würzburg, Core Unit Systems Medicine (SysMed). All samples were sequenced at equimolar ratios.

IDT Custom Primers - ChIPmentation

	I5_n	I7_n
H3K4me3 - Mock Rep. 1	AATGATACGGCGACCACCGAGATCTACAC**AACCGTTC**TCGTCGGCAGCG*T*C	CAAGCAGAAGACGGCATACGAGAT**CTAGCTCA**GTCTCGTGGGCTC*G*G
H3K4me3 - HSV-1 Rep. 1	AATGATACGGCGACCACCGAGATCTACAC**TCAGGCTT**TCGTCGGCAGCG*T*C	CAAGCAGAAGACGGCATACGAGAT**ATCATGCG**GTCTCGTGGGCTC*G*G
H3K4me3 - Mock Rep. 2	AATGATACGGCGACCACCGAGATCTACAC**AACCGTTC**TCGTCGGCAGCG*T*C	CAAGCAGAAGACGGCATACGAGAT**TATGGCAC**GTCTCGTGGGCTC*G*G
H3K4me3 - HSV-1 Rep. 2	AATGATACGGCGACCACCGAGATCTACAC**TAACCGGT**TCGTCGGCAGCG*T*C	CAAGCAGAAGACGGCATACGAGAT**GTCCTAAG**GTCTCGTGGGCTC*G*G

* indicates phosphorothioated bases

Unique barcodes for each primer are indicated in bold
